# Arid1a deficiency sensitises pancreatic cancer to fatty acid synthase inhibition

**DOI:** 10.1002/ctm2.70394

**Published:** 2025-07-07

**Authors:** Tzu‐Lei Kuo, Ya‐Chin Hou, Yan‐Shen Shan, Li‐Tzong Chen, Wen‐Chun Hung

**Affiliations:** ^1^ National Institute of Cancer Research, National Health Research Institutes Tainan Taiwan; ^2^ Institute of Clinical Medicine, School of Medicine, National Cheng Kung University Tainan Taiwan; ^3^ Department of Surgery National Cheng Kung University Hospital, College of Medicine, National Cheng Kung University Tainan Taiwan; ^4^ Division of Hematology & Oncology, Department of Internal Medicine Kaohsiung Medical University Hospital Kaohsiung Taiwan; ^5^ Faculty of Medicine College of Medicine, Kaohsiung Medical University Kaohsiung Taiwan; ^6^ Department of Pharmacy College of Pharmacy, Kaohsiung Medical University Kaohsiung Taiwan; ^7^ Department of Biological Science and Technology National Yang Ming Chiao Tung University Hsinchu Taiwan

**Keywords:** ARID1A, fatty acid metabolism, fatty acid synthase (FASN), K‐ras mutation, pancreatic cancer, tumour microenvironment

## Abstract

**Background:**

Mutations in the *AT‐rich interactive domain‐containing protein 1A (ARID1A)* gene are frequently found in pancreatic cancer. However, the contribution of ARID1A inactivation to pancreatic tumorigenesis remains unclear. Previous work showed that depletion of Arid1a at early developmental stages induces metabolic disturbance and diabetes mellitus in mice.

**Methods and Results:**

In this study, we generated a genetically engineered mouse model harboring both *K‐ras* mutation and *Arid1a* depletion (KAR mice). We found that the combination of these two genetic alterations induces pancreatic tumor formation. Compared to tumors in *K‐ras* and *Tp53*‐mutant mice (KPC mice), KAR tumors showed increased immune cell infiltration and reduced stromal activation. Transcriptomic analysis revealed significant upregulation of fatty acid metabolism and *fatty acid synthase (FASN)* in KAR tumors, with ARID1A directly regulating *Fasn* expression. Pharmacological inhibition of FASN reduced tumor cell viability and slowed tumor progression in vivo. Analysis of clinical datasets showed an inverse correlation between ARID1A and FASN expression, with high FASN levels predicting worse patient survival.

**Conclusion:**

ARID1A deficiency promotes fatty acid metabolism to accelerate pancreatic tumorigenesis. FASN is a potential therapeutic target for ARID1A‐deficient pancreatic cancer.Mutations in AT‐rich interactive domain‐containing protein 1A (*ARID1A*) gene are frequently found in pancreatic cancer. However, the contribution of ARID1A inactivation to pancreatic tumourigenesis is not well‐characterised. Previously, we generated genetically engineered mice with specific depletion of *Arid1a* gene in the pancreas and found that depletion of *Arid1a* at early developmental stage induced metabolic disturbance and diabetes mellitus. In this study, we established a mouse model with *K‐ras* mutation and *Arid1a* depletion (KAR mice) in the pancreas and showed that the combination of these two genetic alterations induced pancreatic tumour formation. Compared to the tumours developed in mice with *K‐ras* mutation and *Tp53* deficiency (KPC mice), KAR tumours exhibited increased immune cell infiltration and reduced stromal activation. Our results demonstrated a significant upregulation of fatty acid metabolism and fatty acid synthase (FASN) in the KAR tumours, with ARID1A directly regulating *FASN* expression. Inhibition of FASN by chemical inhibitor reduced tumour cell viability and slowed tumour progression in mice. Clinical data revealed a negative correlation between ARID1A expression and FASN, with high FASN levels associated with worse patient survival. Collectively, ARID1A deficiency upregulates fatty acid metabolism to accelerate pancreatic tumourigenesis and FASN is a potential therapeutic target for ARID1A‐deficient pancreatic cancer.

**Key points:**

- ARID1A mutations drive metabolic reprogramming in pancreatic cancer.
- Co‐occurrence of K‐ras mutation and Arid1a loss induces tumor formation with distinct immune microenvironment features.
- FASN is upregulated by ARID1A deficiency and its inhibition suppresses tumor growth.
- Targeting FASN may benefit patients with ARID1A‐deficient pancreatic cancer.

## Graphical abstract

1. Combination of Kras activation and Arid1a loss induces pancreatic cancer in mice.
2. ARID1A deficiency enhances immune cell infiltration and lipid metabolism in pancreatic cancer.
3. Fatty acid synthase (FASN) is a druggable target in ARID1A‐deficient pancreatic cancer.

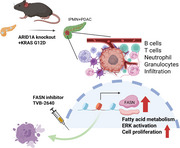

## 1 INTRODUCTION

Pancreatic cancer is a devastating disease characterised by a low 5‐year survival rate. Unfortunately, surgical resection, which offers a chance for a cure, is only feasible for approximately 20% of patients.^1^ For patients with locally advanced or metastatic pancreatic tumours, gemcitabine (GEM)‐based chemotherapy is a standard treatment option.^2^ Although initial response rates to GEM are promising, many patients develop resistance to the therapy after prolonged treatment. Therefore, there is an urgent need to identify new molecular targets for the treatment of this deadly disease.

To gain insights into the dysregulated genes involved in pancreatic tumourigenesis, global genomic profiling has been performed to identify genetic alterations in human pancreatic tumours. The most highly mutated genes are *K‐RAS*, *TP53*, *SMAD4* and *CDKN2A*.^3^, ^4^, ^5^ Additionally, mutations in subunit genes of the switch/sucrose‐non‐fermentable (SWI/SNF) complex have been found in 20% of cases, with *ARID1A* being the most frequently mutated one.^6^ In addition to mutation, loss of *ARID1A* expression is associated with worse clinical outcomes in different cancers including pancreatic cancer.^7^, ^8^, ^9^ The mammalian SWI/SNF complex is a chromatin remodelling enzyme that is composed of more than 15 components encoded by different genes.^10^ This complex modulates chromatin organisation and nucleosome positioning and collaborates with different transcription factors like AP‐1 and FOXA1 to regulate the expression of downstream target genes.^11^, ^12^ ARID1A is the DNA binding subunit in the SWI/SNF complex and is a critical component for the recruitment of the complex to chromatin. Despite high frequency of mutation and reduced expression, the precise role of *ARID1A* in pancreatic tumourigenesis remains elusive.

One pioneer study reported that pancreas‐specific deletion of *Arid1a* in mice induced dilation of pancreatic ducts and reduced expression of SOX9 which led to de‐differentiation of pancreatic ductal cells.^13^ Two subsequent studies demonstrated that *Arid1a* loss resulted in pancreas atrophy, mucinous cyst formation, disruption of acinar cell homeostasis and development of pre‐malignant lesions.^14^, ^15^ When activated *K‐ras* gene was introduced into the *Arid1a*‐depleted mice, all three studies showed the development of pancreatic tumours in the mice. These results suggested that *Arid1a* plays a tumour‐suppressive role in pancreatic cancer. However, a paradoxical role of *Arid1a* in restraining the growth of pancreatic cancer cells in vitro and in vivo has also been reported.^16^ Therefore, more investigations are needed to clarify the physiological and pathological functions of *Arid1a* in the pancreas.

Recently, we generated genetically engineered mice with specific depletion of *Arid1a* gene in the pancreas.^17^ Our results demonstrated that depletion of *Arid1a* at early developmental stage impaired islet formation and decreased insulin secretion, which led to metabolic disturbance and diabetes mellitus. Mechanistic study revealed that Arid1a modulates the expression of neurogenin 3, a crucial transcription factor in determining the fate of endocrine progenitor cells, to control islet development. Interestingly, the regulation of endocrine cells by Arid1a could be stage‐ and context‐dependent because a recent study showed that inhibition of Arid1a activated the epidermal growth factor (EGF) and NR4A1 signalling pathways in β cells to potentiate cell regeneration after pancreatectomy, suggesting a role of Arid1a in constraining the proliferation of β cells in adult mice.^18^ We also found that *Arid1a* depletion alone could not induce tumour formation in the pancreas, consisting with the results of previous studies and supporting the notion that the interplay between different dysregulated genes are essential for tumour formation.

Tumour microenvironment orchestrated by different cell types including cancer cells, fibroblasts, endothelial cells, immune cells and neuronal cells plays an important role in promoting cancer progression, chemotherapy resistance and immunotherapy failure.^19^, ^20^, ^21^ However, the dynamic change in tumour microenvironment during cancer progression in different genetically engineered mice has little been reported. In this study, we generated mice with *K‐ras* mutation and *Arid1a* deletion to study their interplay in pancreatic tumourigenesis. Additionally, we did single‐cell RNA sequencing (sc‐RNAseq) to compare the microenvironment of KAR and KPC tumours and revealed the distinct features in tumour microenvironment. Finally, we found an enriched fatty acid metabolic pathway in the KAR tumours and identified a druggable target in the pathway which could be helpful for the treatment of *ARID1A*‐deficient pancreatic cancer.

## 2 RESULTS

### 2.1 Combination of *Arid1a* deletion and *K‐ras* activation induced pancreatic cancer in mice

**FIGURE 1:**
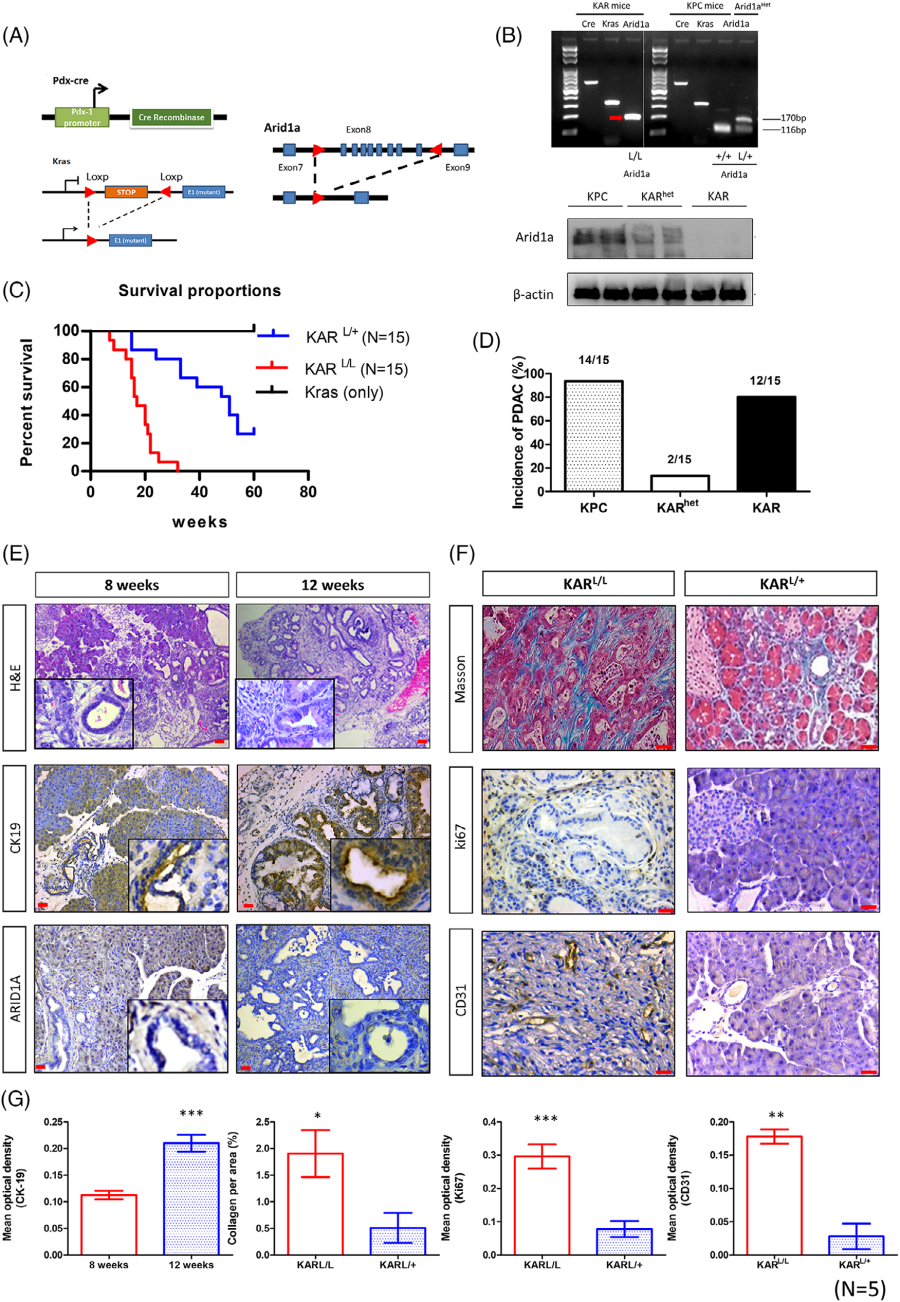
Combination of Arid1a deletion and K‐ras activation induced IPMN and pancreatic cancer in mice. (A) *Pdx‐1Cre*‐mediated deletion of *ARID1A* in the pancreas of Pdx1‐Cre*Kras* ^G12D^ *Arid1a* ^L/L^ (KAR) mice. Structure of the *ARID1A* floxed allele: Loxp sites were inserted into the introns surrounding exons 7 and 9. (B) Genotyping of KPC and KAR mice by polymerase chain reaction (PCR). The Arid1a protein level was analysed by Western blotting. (C) The survival proportions of KAR mice were compared to that of KPC mice (*n* = 15). (D) The incidence of pancreatic tumour formation in KPC, KAR^het^ (ARID1A loss of heterozygosity) and KAR mice (*n* = 15). (E) Representative haematoxylin and eosin (H&E) and CK19 immunohistochemistry (IHC) staining showing the progression of pancreatic intraepithelial neoplasia (PanIN) and carcinoma in the pancreases of KAR mice at different ages (*n* = 5 per time point). (F) CK19 expression in pancreatic lesions of KAR mice collected at 4, 8, 12 and 16 weeks (*n* = 5 per group). (G) Representative Masson's trichrome, CD31 and Ki67 staining of pancreatic tissues from KAR mice (*n* = 5). Scale bars in (E) and (G) represent 20 µm.

Our previous study showed that specific depletion of *Arid1a* in mouse pancreas leads to islet developmental defect and metabolic disturbance.^17^ In order to investigate the role of Arid1a in pancreatic cancer progression, we analysed both genetic and phenotypic features in mouse models with targeted deletions of *Arid1a*. We generated Pdx1‐Cre*Kras* ^G12D^ *Arid1a* ^L/L^ (KAR) mice and compared the phenotypes between KAR and Pdx1‐Cre*Kras* ^G12D^ *Arid1a* ^L/+^ (KAR^het^) mice (Figure 1A). We used polymerase chain reaction (PCR) to confirm the *Arid1a* genotype, ensuring the presence of both *Arid1a* ^L/L^ and *Arid1a* ^L/+^ genotypes (Figure 1B). In addition, the reduction of Arid1a protein in heterozygous and homozygous mice was confirmed by Western blotting (Figure 1B). The KAR mice exhibited significantly shorter survival compared to KAR^het^ mice (*p* < .001), with a median survival time of 17 weeks (Figure 1C). The incidence of pancreatic tumour formation in KPC and KAR mice was 93% and 80% respectively at 16 weeks of age (Figure 1D). Interestingly, only 13% of the KAR^het^ mice developed pancreatic tumour at the same age, suggesting a dosage‐dependent effect of *Arid1a* in inhibiting tumourigenesis. Pathological examination demonstrated the presence of low‐grade pancreatic intraepithelial neoplasia (PanIN) lesions emerging around 8 weeks after birth. High‐grade PanINs and invasive pancreatic tumours were observed between 12 and 16 weeks of age. Increased cytokeratin 19 (CK19) staining was observed in these neoplastic lesions (Figure 1E,G). Desmoplasia, examined by Masson's trichrome staining, was more pronounced in KAR tumours than in KAR^het^ tumours, corresponding with a significantly higher collagen‐positive area in the KAR tumours (Figure 1F,G). Additionally, the KAR tumours displayed increased vascularity, as confirmed by CD31 staining, and a higher number of Ki67‐positive cancer cells, indicating greater proliferation, compared to the KAR^het^ tumours (Figure 1F,G). These results suggest that the KAR tumours are more aggressive than the KAR^het^ tumours, indicating a dosage‐dependent effect of Arid1a in inhibiting tumourigenesis. We also found that Arid1a is expressed in normal pancreases and the pancreases collected from KPC mice (Figure S1).

### 2.2 Dynamic change in tumour microenvironment during cancer progression in the KAR mice

**FIGURE 2:**
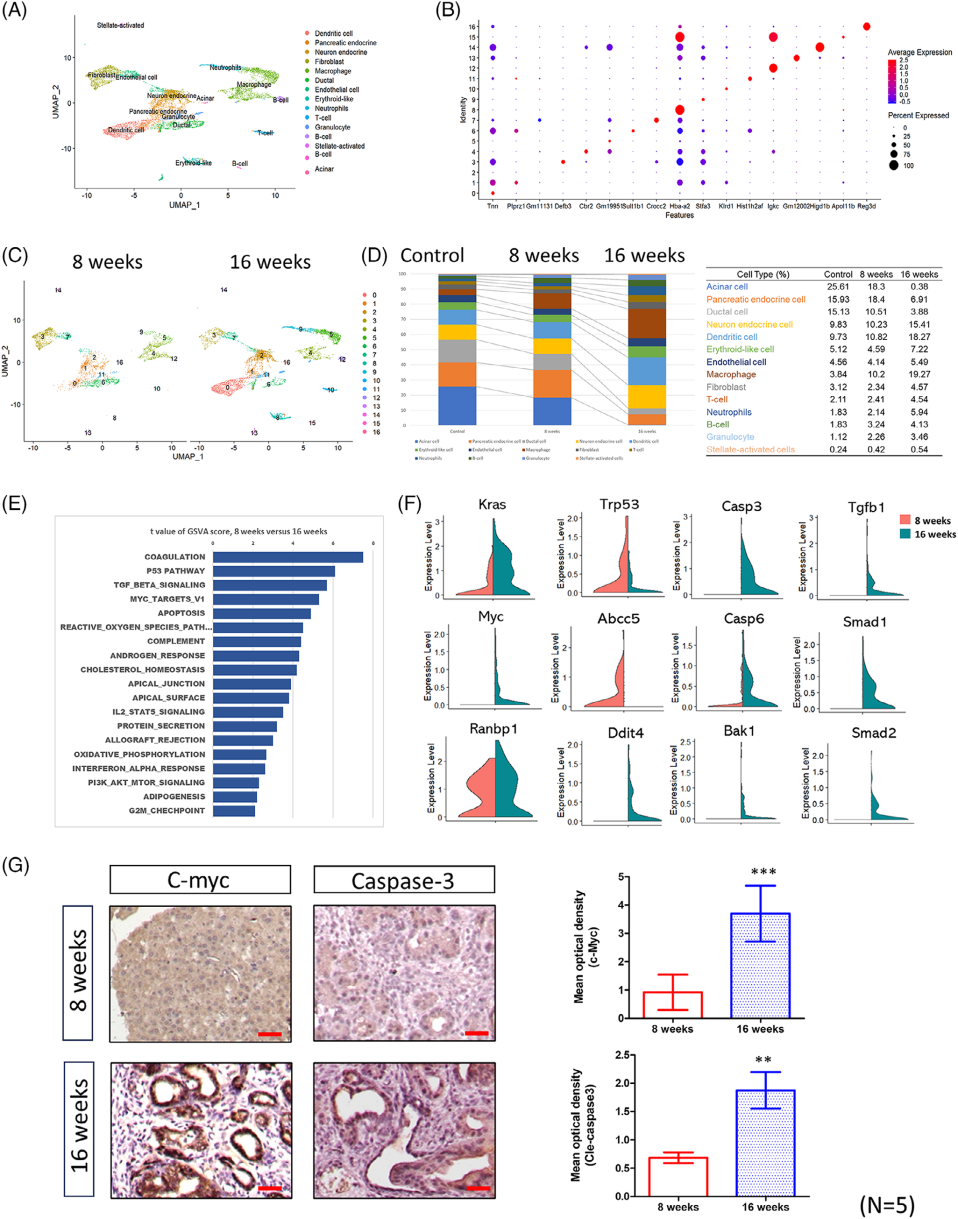
Single‐cell RNA sequencing (sc‐RNAseq) analysis of pancreases collected from normal and KAR mice at different time points. (A) UMAP plot showing 14 distinct cell types identified from the integrated sc‐RNAseq dataset of control mice and KAR mice at 8 and 16 weeks of age. Cell types were annotated based on the expression levels of representative canonical marker genes. (B) Dot plot displaying differentially expressed marker genes used to distinguish major cell populations. The colour intensity reflects average expression, and dot size represents the percentage of cells expressing each gene within a given cluster. (C) UMAP plot of pancreatic cells from KAR mice at 8 and 16 weeks of age, showing 17 transcriptionally distinct clusters identified through dimensionality reduction analysis. (D) The percentages of cell types in the pancreases of normal and KAR mice at different ages. (E) Gene set variation analysis (GSVA) analysis showed the enriched pathways in the pancreatic cells of 16‐week‐old KAR mice when compared to that of 8‐week‐old KAR mice. (F) Violin plot showed the expression of genes in the proliferation, fibrosis and cell death pathways enriched in 8‐week‐old (orange colour) or 16‐week‐old (green colour) KAR mice. (G) Immunohistochemistry (IHC) staining confirmed the increased expression of c‐Myc and cleaved caspase‐3 in the pancreases of 16‐week‐old KAR mice (*n* = 5).

Having established that *Arid1a* deletion promotes pancreatic tumourigenesis, we next sought to investigate how these genetic changes influence the tumour microenvironment during cancer progression. To comprehensively analyse dynamic changes in the tumour microenvironment, we performed sc‐RNAseq on pancreatic tissues from KAR mice at 8 and 16 weeks of age, as well as from control mice. Integrated analysis of these datasets identified 14 distinct cell types across all samples. Figure 2A presents the uniform manifold approximation and projection (UMAP) plot generated from the combined dataset, illustrating the distribution of all identified cell populations from both control and KAR pancreases. We annotated cell types by using classic signatures reported in a previous study.^22^ The differentially expressed genes (DEGs) across cell types are displayed in a dot plot (Figure 2B), highlighting transcriptional distinctions among the major populations. We pre‐processed the sc‐RNAseq data through gene filtering, normalisation and principal component analysis, ultimately identifying 17 distinct clusters in tumours collected from 8‐ and 16‐week‐old mice (Figure 2C). As shown in Figure 2D, acinar cells, endocrine cells and ductal cells were the most abundant cell types in normal pancreas. However, their abundances were significantly decreased during tumour development. On the contrary, the percentages of immune cells including dendritic cells (9.73%–18.27%), macrophages (3.84%–19.27%), T cells (2.11%–4.54%), neutrophils (1.83%–5.94%) and granulocyte (1.12%–3.46%) were significantly increased. We focused on the gene expression profile in pancreatic ductal cells and performed gene set variation analysis (GSVA) to identify the altered pathways. Several pathways related to proliferation (Myc target and G2/M checkpoint), fibrosis (TGF‐β signalling), cell death (p53 and apoptosis) and cellular metabolism (cholesterol homeostasis, adipogenesis and oxidative phosphorylation) were changed in the 16‐week tumours when compared with the 8‐week precancer lesions (Figure 2E). The expressions of genes in these pathways like *K‐ras*, *Myc*, *Tp53*, caspases, *Smad1/2* were significantly altered (Figure 2F). Furthermore, we used immunohistochemistry (IHC) to confirm the expression of c‐Myc and caspase‐3, showing consistency with the scRNA data, and observed that both c‐Myc and caspase‐3 were highly expressed in late‐stage tumours (Figure 2G). Thus, during the development of KAR tumours, cell populations and gene expression profiles in the microenvironment were dynamically changed.

### 2.3 Distinct features in the microenvironment of KPC and KAR tumours

**FIGURE 3:**
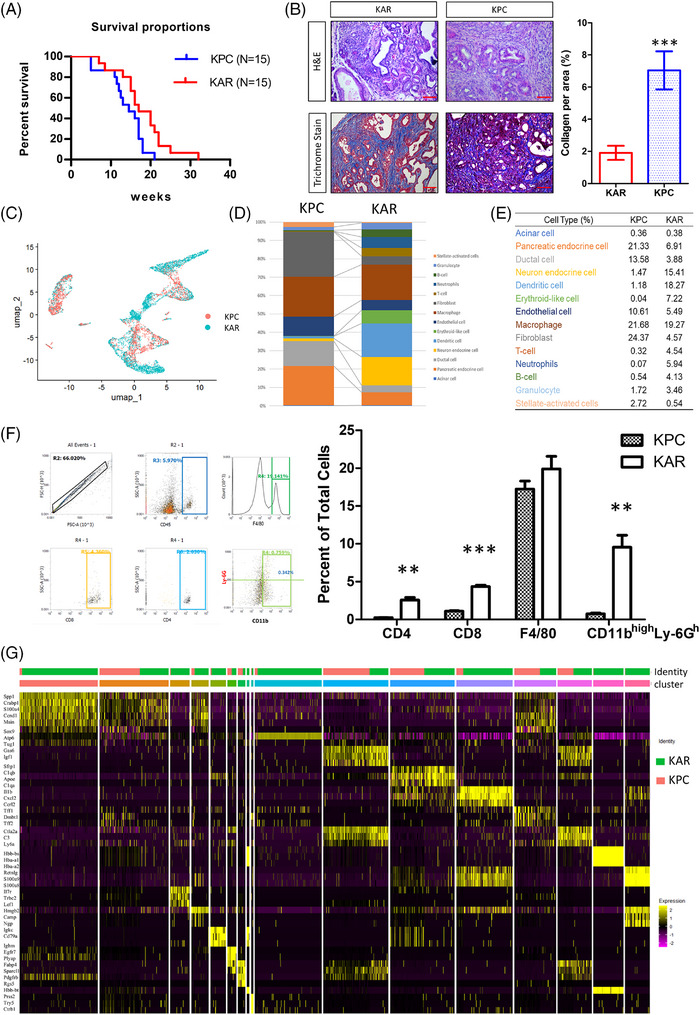
Distinct features in the microenvironment of KPC and KAR tumours. (A) The survival curve of KPC and KAR mice (*n* = 15). (B) Desmoplasia demonstrated by Masson's trichrome staining in the pancreases of KPC and KAR mice(*n* = 5 per group). (C) UMAP analysis of the cell clusters in the pancreases of KPC and KAR mice. (D) 14 cell types were identified based on the expression level of representative markers. (E) Proportional distribution of cell types in the pancreases of KPC and KAR mice. (F) Flow cytometric analysis confirmed the increase of CD4^+^, CD8^+^ and neutrophils (CD11b^high^Ly‐6G^high^) in the KAR tumours. (G) Heatmap presenting the expression levels of marker genes in different type cells and the top three highly expressed genes were shown.

To understand how *Arid1a* deletion and *K‐ras* mutation uniquely shape tumour characteristics, we compared KAR tumours to the well‐established KPC model of pancreatic cancer. The KAR mice lived longer than the KPC mice (*p* = .027) and the median survival of KAR and KPC mice was 17 weeks and 14.5 weeks (Figure 3A). Morphological examination showed that the KAR tumours were more differentiated and less fibrotic than the KPC tumours (Figure 3B). To further elucidate the distinct features of tumour microenvironment in these two models, we performed sc‐RNAseq to compare cellular populations and gene expression profiles in KAR and KPC tumours. As shown in Figure 3C, the populations of cells in these two types of tumours were significantly different. Fourteen cell clusters in the KAR and KPC tumours were annotated (Figure 3D). Compared to the KPC tumours, KAR tumours have more immune cell infiltration (4.13% vs. .54% of B cells; 3.46% vs. 1.72% of granulocytes; 4.54% vs. .32% of T cells and 5.94% vs. .07% of neutrophils; Figure 3E). Another distinct feature is the low abundance of fibroblasts in the KAR tumours (4.57%) when compared with that of KPC tumours (24.37%), agreeing with the results of Masson's trichrome staining in tumour tissues (Figure 3B). Flow cytometric analysis confirmed the increase of CD4^+^, CD8^+^ and neutrophils (CD11b^high^Ly‐6G^high^) in the KAR tumours while the percentage of macrophages was similar (Figure 3F). To further examine the differences between KAR and KPC tumours, we re‐clustered gene expression profiles within cell types and showed the top three highly expressed genes in each cell type (Figure 3G). The results demonstrated significant changes in gene expression profiles, suggesting the heterogeneity during cancer progression.

### 2.4 Enhancement of fatty acid metabolism and upregulation of *Fasn* expression in KAR tumours

**FIGURE 4:**
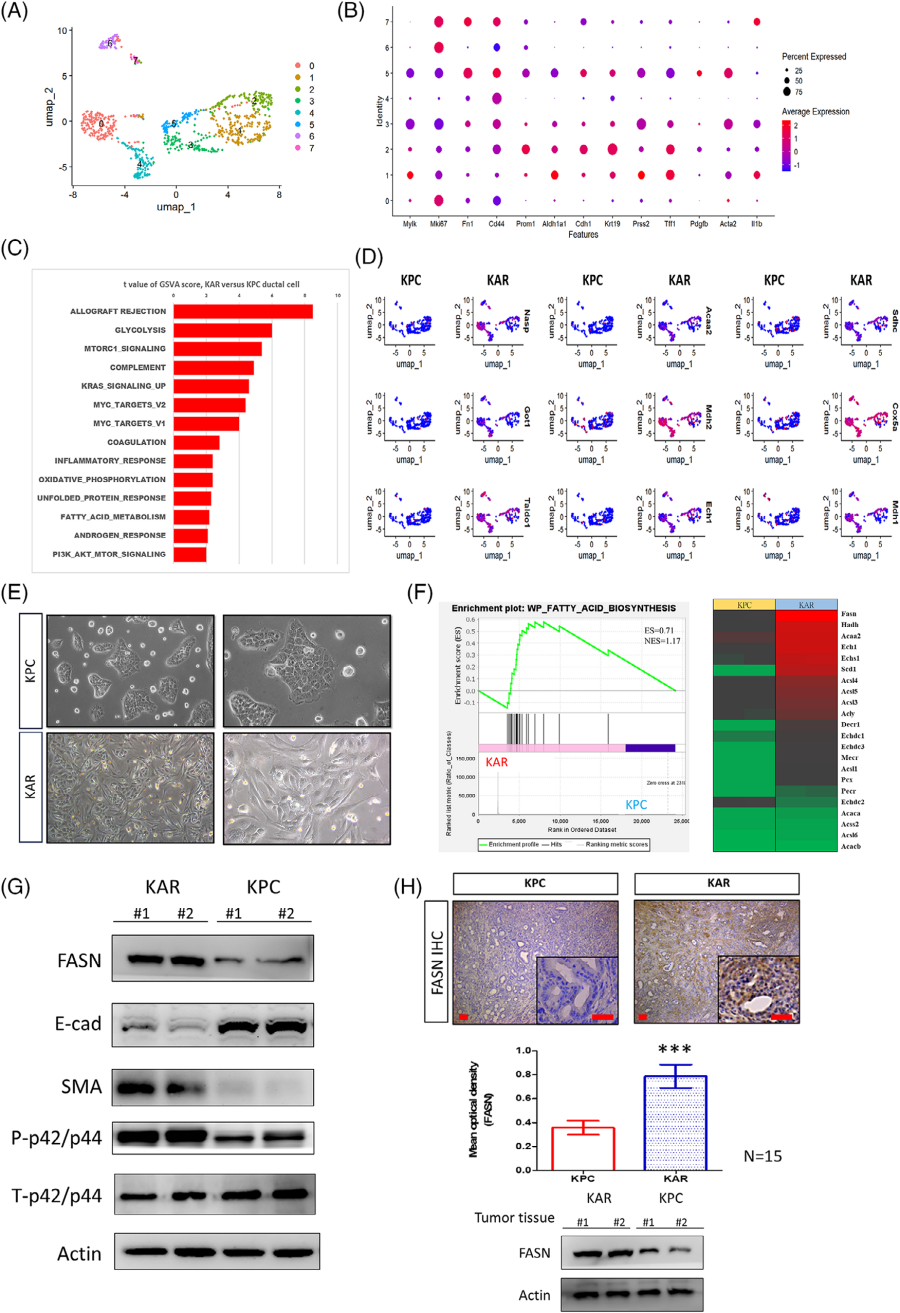
Enhancement of fatty acid metabolism and upregulation of fatty acid synthase (FASN) expression in cancer cells in the KAR tumours. (A) UMAP plots demonstrated the nine cancer cell subpopulations in subcluster analysis. (B) The differentially expressed marker genes were presented in a dot plot. (C) Gene set variation analysis (GSVA) showed the enriched pathways in cancer cells expressing ductal cell markers in the KAR and KPC tumours. (D) Upregulation of metabolic genes in glycolysis, fatty acid metabolism and oxidative phosphorylation in the KAR cancer cells when compared to the KPC cancer cells. (E) Morphology of the isolated KAR and KPC cancer cells observed under a light microscope. Scale bars represent 50 µm. (F) Gene Set Enrichment Analysis (GSEA) revealed the upregulation of fatty acid biosynthesis pathway in the KAR cancer cells. Heatmap of genes in fatty acid biosynthesis pathway were shown. (G) Immunoblotting analysis demonstrated the increases of FASN protein and phosphorylated p42/44 in KAR cancer cells. β‐Actin was served as a loading control. Two independent cell clones (#1 and #2) were used in the study to confirm FASN upregulation and extracellular signal‐regulated kinases (ERKs) activation. (H) FASN protein levels in the tumour tissues and the tumour lysates were examined by Immunohistochemistry (IHC) staining and Western blotting. Scale bars represent 50 µm.

To identify potential therapeutic targets, we analysed sc‐RNAseq data of cancer cells from both KPC and KAR tissue samples and subcluster analysis was conducted on the cancer cells through UMAP analysis. Based on gene expression profiles, eight subclasses of cancer cells were defined (Figure 4A). Common markers, such as *Acta2*, *Mki67* and *Krt19*, were expressed in specific subpopulations, confirming the pancreatic cancer cell identity (Figure 4B). GSVA analysis showed significant enhancement of various metabolic pathways including glycolysis, mammalian target of rapamycin (mTOC), fatty acid metabolism and oxidative phosphorylation in the KAR cancer cells when compared to the KPC cancer cells (Figure 4C). The results showed that glycolysis‐related genes, such as *Nasp*, *Got1* and *Taldo1*, were highly expressed in the cancer cells of KAR mice. Additionally, fatty acid metabolism‐related genes, including *Acaa2*, *Mdh2* and *Ech1*, as well as oxidative phosphorylation‐related genes, such as *Sdhc*, *Cox5a* and *Mdh1*, also upregulated in KAR cancer cells (Figure 4D). Next, we established two independent KAR and KPC cancer cell lines from tumour tissues and found that KAR cancer cells showed mesenchymal phenotypes while KPC cancer cells exhibited epithelial morphology (Figure 4E). We specifically focused on fatty acid metabolism and did bulk RNA sequencing to compare gene expression in early passage KAR and KPC cancer cells. Our results confirmed the enhancement of fatty acid biosynthesis pathway and upregulation of *Fasn*, *Ech1*, *Acaa2* and *Hadh*, four metabolic genes in the pathway, in KAR cancer cells (Figure 4F). Increased expression of several target genes in KAR cancer cells was further confirmed by RT‐PCR analysis (Figure S2). *Fasn*, a key enzyme in the endogenous lipogenesis pathway that catalyses the synthesis of long‐chain fatty acid, was highly upregulated in KAR cancer cells (Figure 4G). In agreement with the mesenchymal phenotype observed, KAR cancer cells expressed higher α‐smooth muscle actin (SMA) and lower E‐cadherin when compared with KPC cancer cells (Figure 4G). Immunohistochemical staining and Western blotting also confirmed Fasn overexpression in the KAR tumours (Figure 4H). Together, these findings demonstrated that *Arid1a* deletion in combination with *K‐ras* mutation reprograms cancer cell metabolism and enhances fatty acid biosynthesis, suggesting a novel avenue for targeted therapy.

### 2.5 Fasn is a direct repression target of Arid1a and is an upstream activator of extracellular signal‐regulated kinases (ERKs)

**FIGURE 5:**
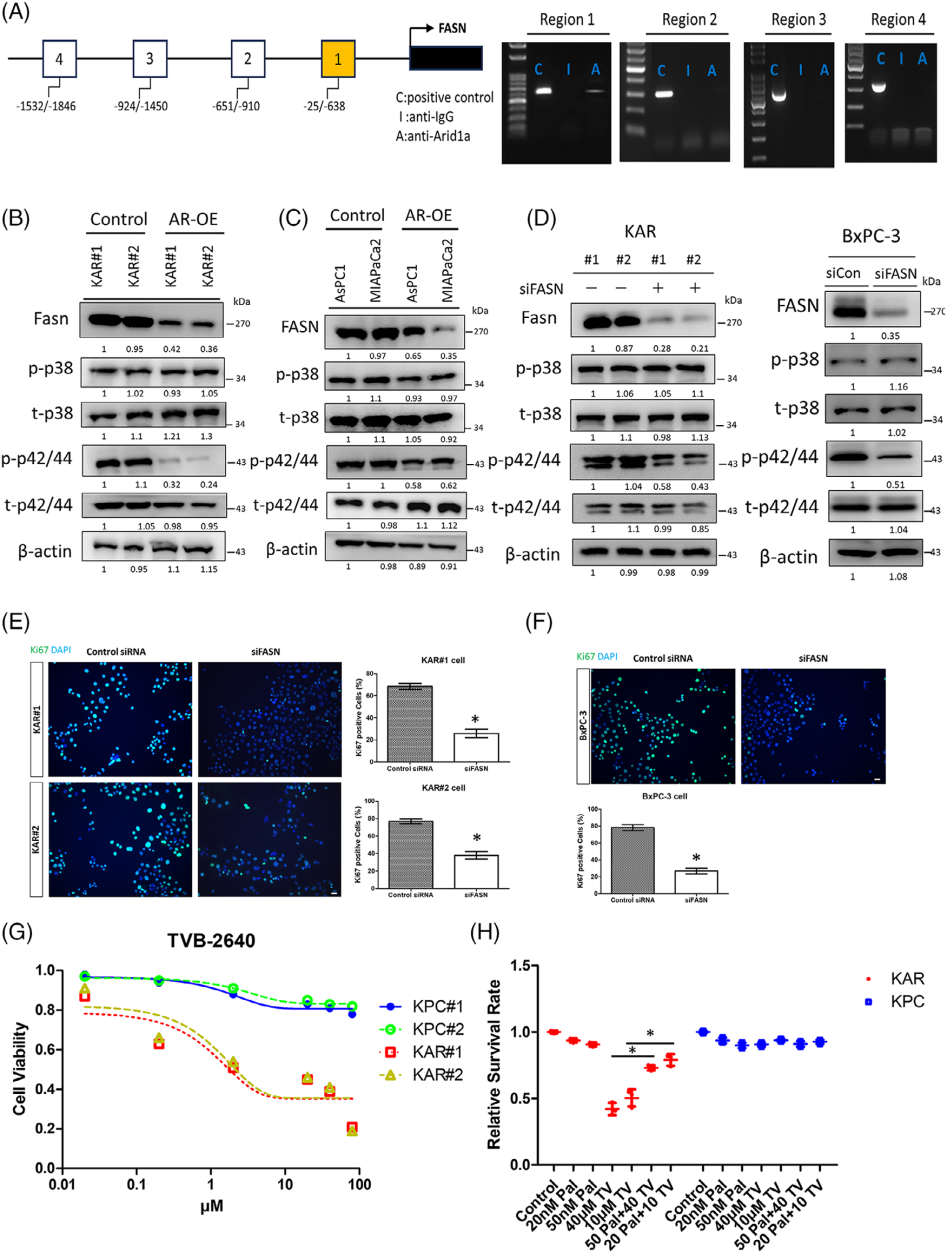
Fatty acid synthase (FASN) is a direct repression target of Arid1a and is an upstream activator of extracellular signal‐regulated kinases (ERKs). (A) The potential Arid1a binding sequences in the FASN gene promoter were identified by bioinformatics prediction and the locations of these sites were indicated. Chromatin immunoprecipitation (ChIP) assays were done to study the interaction of Arid1a with these sites. ChIP‐polymerase chain reaction (PCR) assay demonstrated the binding of Arid1a to a proximal promoter region (‐25/‐638 bp from transcriptional start site) of mouse FASN gene. (B) The activity of various downstream mediators of the FASN signalling pathway was investigated by Western blotting. Re‐expression of *Arid1a* in two independent KAR cancer cell clones (#1 and #2) suppressed the activation of ERK in the cells. (C) Overexpression of *ARID1A* in AsPC‐1 and MiaCaPa‐2 human pancreatic cancer cells decreased FASN expression and ERK activation. (D) Knockdown of FASN by siRNA led to reduction of ERK activation in KAR cancer cells and BxPC3 human pancreatic cancer cells. (E) Two independent clones of the KAR cancer cells were transfected with control or FASN siRNA for 48 h. Fluorescence microscope were used to detect Ki67‐positive cells and the percentages of Ki67‐positive cells were shown. The experiments were repeated three times and the data represent mean ± SEM (*n* = 3). *p < .05. Scale bars represent 20 µm. (F) BxPC3 human pancreatic cancer cells were transfected with control or FASN siRNA for 48 h. Fluorescence microscope were used to detect Ki67‐positive cells. The experiments were repeated three times and the data represent mean ± SEM (*n* = 3). *p < .05. Scale bars represent 20 µm. (G) Two independent clones of KPC and KAR cancer cells were treated with increasing concentrations of TVB‐2640 (20 nM to 80 µM) for 24 h, and cell viability was assessed using the MTT assay. Viability values were normalised to the vehicle‐treated control (DMSO), which was set to 1.0 for each clone. The graph shows the dose‐dependent response of KPC and KAR cells to TVB‐2640 treatment, with KAR cells exhibiting increased sensitivity. (H) The KPC and KAR cancer cells co‐treated without or with palmitic acid and TVB‐2640. The effect of palmitic acid on the viability of TVB‐2640‐treated KPC and KAR cancer cells was compared. Error bars are means ± SEM (*n* = 3), *p < .05. Band intensities were quantified, normalised to first lane.

To investigate whether *Fasn* expression is directly regulated by Arid1a, we performed chromatin immunoprecipitation (ChIP) assay and found that Arid1a binds to a proximal promoter region (−25/−638 bp from the transcriptional start site) of the mouse *Fasn* gene, indicating a direct regulatory role (Figure 5A). Re‐expression of *Arid1a* in KAR cells decreased Fasn protein levels (Figure 5B). A similar effect was observed in the MIAPaCa‐2 and AsPC1 human pancreatic cancer cell lines, which carry *ARID1A* mutations or exhibit low ARID1A expression (Figure 5C). Additionally, Arid1a overexpression significantly reduced the activity of extracellular signal‐regulated kinases (ERKs) in both mouse and human pancreatic cancer cells, while p38 kinase activity remained unchanged (Figure 5B,C). Depletion of Fasn in KAR cancer cells by siRNA led to a significant reduction in ERK activity, while p38 activity was unaffected (Figure 5D). Similarly, inhibition of fatty acid synthase (FASN) in BxPC3 human pancreatic cancer cells also decreased ERK activation (Figure 5D). Furthermore, siRNA‐mediated depletion of FASN significantly inhibited the proliferation of KAR and BxPC3 cancer cells, as demonstrated by decreased Ki67 staining (Figure 5E,F). To assess the sensitivity of KAR cells to FASN inhibition, we treated KAR and KPC cells with TVB‐2640, a FASN inhibitor in phase 2 clinical trials. TVB‐2640 effectively suppressed the growth of KAR cancer cells in a dose‐dependent manner (Figure 5G). Conversely, the proliferation of KPC cells was not significantly affected. Inhibition of FASN by siRNA also suppressed the growth of mouse (KAR#1 and KAR#2) and human (BxPC3) pancreatic cancer cells with ARID1A loss (Figure S3). Supplementing with palmitic acid, the end‐product of FASN‐catalysed lipid biosynthesis, reversed the TVB‐2640‐induced growth inhibition in KAR cancer cells, but had no effect on KPC cells (Figure 5H). Our data suggested that FASN overexpression induced by ARID1A loss may promote ERK activity to stimulate the proliferation of pancreatic cancer cells.

**FIGURE 6:**
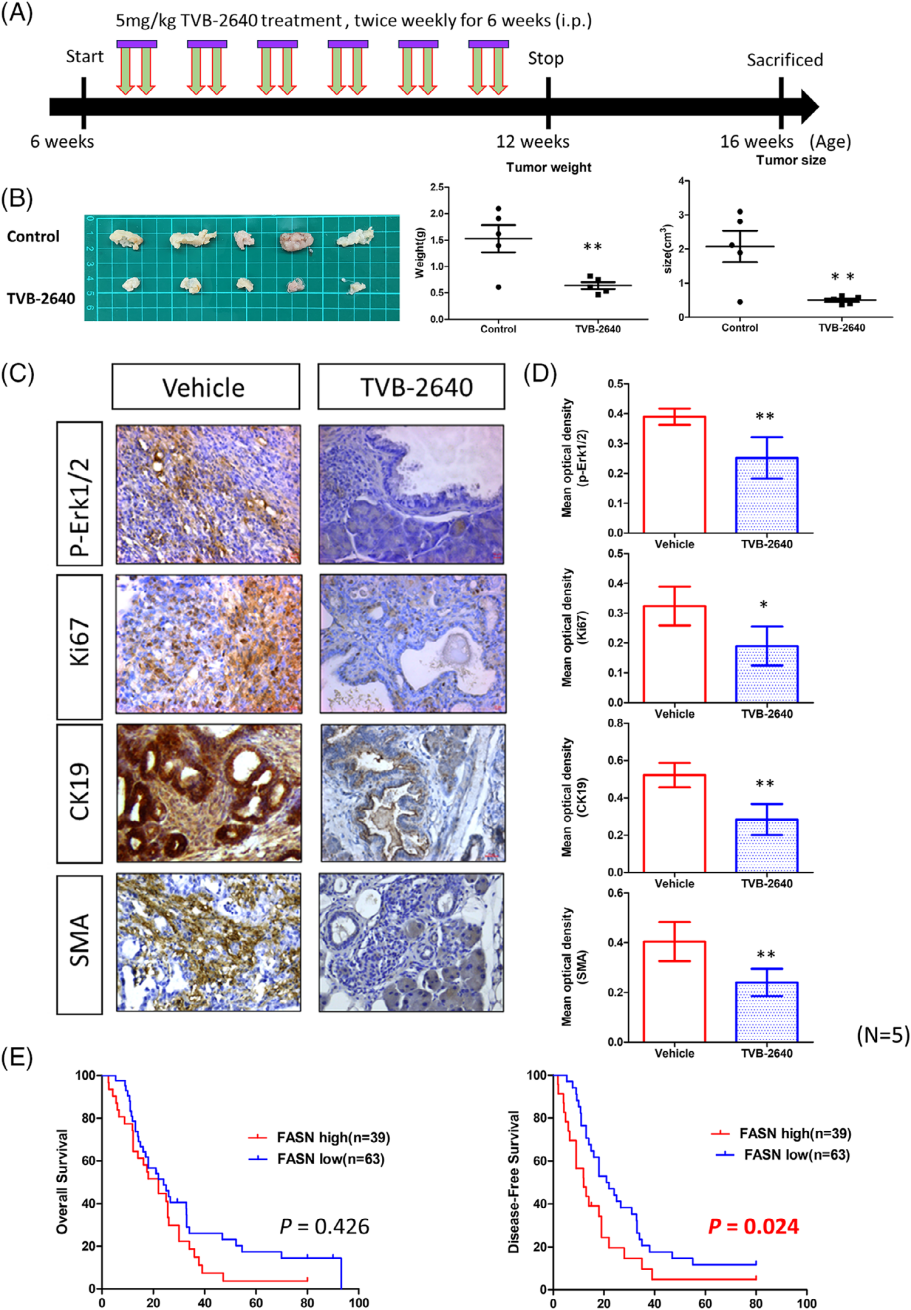
Inhibition of fatty acid synthase (FASN) signalling blocks tumour formation in KAR mice. (A) Scheme showed the protocol of TVB‐2640 treatment in the KAR mice. The 6‐week‐old KAR mice were given TVB‐2640 (5 mg/kg) or vehicle by intraperitoneal injection twice a week for 6 weeks. (B) The mice (*n* = 5) were sacrificed 4 weeks after the end of TVB‐2640 treatment. Tumour size and tumour weight of the control and TVB‐2640 groups were compared. (C)Tumour tissues of the control and TVB‐2640‐treated mice were subjected to immunohistochemistry (IHC) staining to detect the expression of p‐ERK1/2, Ki67, CK19 and smooth muscle actin (SMA). Scale bars represent 50 µm. (D) The signal intensities of p‐ERK1/2, Ki67, CK19 and SMA in the tumour tissues of control and TVB‐2640‐treated groups were quantified (*n* = 15, three image fields for each sample and five samples from each group were used for analysis). (E) Kaplan–Meier survival curves comparing disease‐free survival between patients with high versus low FASN expression (*n* = 102). Patients with high FASN expression exhibited significantly shorter disease‐free survival (median DFS: 11.8 vs. 18.2 months), supporting the role of FASN as a potential prognostic marker in pancreatic cancer. Statistical significance was determined using the log‐rank test.

Given the increased sensitivity of KAR cells to FASN inhibition in vitro, we investigated the anti‐cancer effects of TVB‐2640 in vivo in KAR mice. Because low‐grade PanIN lesions were detected in the KAR mice at 8 weeks of age, we began TVB2640 treatment (5 mg/kg, twice a week) on 6‐week‐old mice. After 6 weeks, drug administration was stopped. We harvested the pancreases from mice 1 month after final treatment (Figure 6A). As shown in Figure 6B, tumour sizes and tumour weights in the TVB2640‐treated group were significantly decreased. In addition, ERK activation and Ki67‐positive cancer cells were dramatically reduced in the TVB2640‐treated group (Figure 6C,D). Histological examination of lesions from TVB2640‐treated mice showed decreased CK19^+^ tumour cell population, suggesting the treatment decreased the development to pancreatic ductal carcinoma (Figure 6C,D). Moreover, the expression of SMA was significantly reduced in the TVB2640‐treated tumours. In addition, ERK activity was significantly inhibited by TVB2640 treatment (Figure S4). These data suggested that Fasn is a vulnerable therapeutic target in Arid1a‐deficient pancreatic cancer.

To confirm the clinical relevance of our findings, we analysed *ARID1A* and *FASN* expression in a pancreatic cancer cohort from The Cancer Genome Atlas (TCGA) dataset and observed a negative correlation between ARID1A and FASN expression (Figure S5). We next used a pancreatic cancer patient cohort (*n* = 102) collected in our hospital to test the association between ARID1A and FASN with clinicopathological features of patients. High expression of FASN in ARID1A‐deficient patients was confirmed by IHC staining (Figure S6). Our results showed that ARID1A expression was not correlated clinicopathological parameters (Table 1). Because mutations are more frequently found than downregulation in the *ARID1A* gene in pancreatic cancer, the association needs to be re‐examined after checking the mutation status of *ARID1A* gene in the patients. On the contrary, high FASN expression was significantly correlated with poor tumour differentiation, high body mass index (BMI) and increased cancer cachexia (Table 1). ARID1A expression was also not associated with patient's survival (Figue S7). However, patients with high FASN expression had significantly worse overall and disease‐free survival, underscoring FASN as a potential prognostic factor in pancreatic cancer (Table 2 and Figure 6E). The median disease‐free survival was 18.2 versus 11.8 months in low‐ and high‐FASN groups. These findings highlight the potential of FASN as a therapeutic target in Arid1a‐deficient pancreatic cancers and support further exploration of FASN inhibitors as a treatment strategy in these cases.

**TABLE 1:** Comparison of patient characteristics based on ARID1A and fatty acid synthase (FASN) (*n* = 102).

	ARID1A			FASN		
Variable	Low	High	*p* value	Low	High	*p* value
**Age**						
≦65 years	25 (48.1)	21 (42.0)	.269	32 (50.8)	14 (35.9)	.071
>65 years	27 (51.9)	29 (58.0)		31 (49.2)	25 (64.1)	
**Gender**						
Male	25 (48.1)	26 (52.0)	.346	34 (54.0)	17 (43.6)	.154
Female	27 (51.9)	24 (48.0)		29 (46.0)	22 (56.4)	
**Stage**						
I	8 (15.4)	11 (22.0)	.089	11 (17.5)	8 (20.5)	.472
II	37 (71.2)	26 (52.0)		40 (63.5)	23 (59.0)	
III	4 (7.7)	10 (20.0)		8 (12.7)	6 (15.4)	
IV	3 (5.8)	3 (6.0)		4 (6.3)	2 (5.1)	
**Tumour location**						
Head/neck/uncinate process	42 (80.8)	35 (70.0)	.103	50 (79.4)	27 (69.2)	.124
Body/tail	10 (19.2)	15 (30.0)		13 (20.6)	12 (30.8)	
**Tumour grade**						
Well diff.	6 (11.5)	5 (10.0)	.399	4 (6.3)	7 (17.9)	**.005**
Moderately diff.	40 (76.9)	37 (74.0)		54 (85.7)	23 (59.0)	
Poorly diff.	6 (11.5)	8 (16.0)		5 (7.9)	9 (23.1)	
**Tumour size**						
≦3 cm	24 (46.2)	25 (50.0)	.349	33 (52.4)	16 (41.0)	.133
>3 cm	28 (53.8)	25 (50.0)		30 (47.6)	23 (59.0)	
**Resection margin**						
Negative	42 (80.8)	43 (86.0)	.240	53 (84.1)	32 (82.1)	.393
Positive	10 (19.2)	7 (14.0)		10 (15.9)	7 (17.9)	
**Vascular invasion**						
Absent	25 (48.1)	23 (46.0)	.417	29 (46.0)	19 (48.7)	.396
Present	27 (51.9)	27 (54.0)		34 (54.0)	20 (51.3)	
**Lymphatic invasion**						
Absent	25 (48.1)	23 (46.0)	.417	29 (46.0)	19 (48.7)	.396
Present	27 (51.9)	27 (54.0)		34 (54.0)	20 (51.3)	
**Perineural invasion**						
Absent	6 (11.5)	3 (6.0)	.162	6 (9.5)	3 (7.7)	.376
Present	46 (88.5)	47 (94.0)		57 (90.5)	36 (92.3)	
**BMI**						
≦18.5 kg/m2	6 (11.5)	2 (4.0)	.086	7 (11.1)	1 (2.6)	**.019**
18.6–24 kg/m^2^	21 (40.4)	28 (56.0)		34 (54.0)	15 (38.5)	
>24 kg/m^2^	25 (48.1)	20 (40.0)		22 (34.9)	23 (59.0)	
**Cancer cachexia**						
Absent	20 (38.5)	14 (28.0)	.132	25 (39.7)	9 (23.1)	**.042**
Present	32 (61.5)	36 (72.0)		38 (60.3)	30 (76.9)	

Abbreviations: BMI, body mass index; diff., differentiation.

The bold value indicates *p* < .05.

**TABLE 2:** The association of ARID1A and fatty acid synthase (FASN) with overall survival and disease‐free survival.

Variable	Patients (%)	Overall survival		Disease‐free survival	
		Median (months)	*p* value	Median (months)	*p* value
**ARID1A**					
Low	52 (51.0)	24.778	.938	14.624	.4
High	50 (49.0)	22.412		14.722	
**FASN**					
Low	63 (61.8)	28.459	.426	18.239	**.024**
High	39 (38.2)	20.769		11.798	

The bold value indicates *p* < .05.

## 3 DISCUSSION

Because *ARID1A* inactivation is frequently found in pancreatic cancer, the identification of vulnerable genes in *ARID1A*‐deficient pancreatic cancer by using different experimental approaches has been intensively performed in recent years and several dysregulated pathways have been suggested to be potential therapeutic targets. First, ARID1A loss in cancer cells impairs the DNA damage checkpoint and increases sensitivity to poly ADP‐ribose polymerase (PARP) inhibitors.^23^ Extended from the study, ARID1A‐deficient cancer cells were found to be susceptible to Ataxia‐telangiectasia and rad3‐related protein kinase (ATR) inhibitors because these cancer cells show defects in topoisomerase 2A and cell cycle regulation, leading to a dependency in ATR function.^24^ Third, activation of the PI3K/AKT signalling was frequently observed in pancreatic cancer cells with ARID1A loss.^25^ Mechanistic study revealed that ARID1A activates the expression of PI3K‐interacting protein 1 to suppress PI3K activation and ARID1A deficiency enhances the PI3K/AKT/mTOC pathway.^26^ In addition, PI3K/AKT inhibitors have been shown to enhance radiosensitivity of ARID1A‐deficienct pancreatic cancer cells.^27^ Fourth, ARID1A‐depleted cancer cells are more sensitive to the enhancer of zeste homolog 2 methyltransferase (EZH2) inhibitors because ARID1A and EZH2 are functionally antagonistic in the control of gene expression.^28^ In this study, we identified a new therapeutic target FASN in ARID1A‐deficient pancreatic cancer. Our results demonstrated that ARID1A directly bind to the FASN promoter to repress its expression and ARID1A inactivation enhances FASN expression and ERK activation (Figure 4). In addition, inhibition of FASN by chemical inhibitors or siRNA knockdown attenuated ERK activation and proliferation in ARID1A‐defecient pancreatic cancer cells. FASN inhibitor TVB2640 also effectively suppresses tumour growth in the KAR mice. Moreover, FASN expression is highly expressed in *ARID1A*‐low tumours and is associated with worse survival in pancreatic cancer patients. Single agent treatment or combined therapy with TVB2640 is now under different phases of clinical trials for the treatment of human diseases including cancers. The application of TVB2640 in the treatment of ARID1A‐deficient pancreatic cancer patients warrant further investigation.

Our findings demonstrate a clear genetic dosage effect of ARID1A loss in pancreatic cancer, as homozygous deletion (KAR^L/L^) led to more aggressive tumour progression and shorter survival compared to heterozygous deletion (KAR^L/+^). Several large‐scale human cancer studies suggest that hemizygous loss of ARID1A is sufficient to drive tumour development in multiple cancers. We agree that the genetic dosage effect of ARID1A and its biological consequences can vary depending on tissue context, tumour type and species‐specific compensatory mechanisms. In addition to mutations, ARID1A gene could be inactivated by epigenetic inactivation, which has not been extensively investigated. We think the discrepancy between our mouse model and human studies may be due to several factors. First, species‐specific differences in epigenetic regulation and chromatin remodelling compensation could influence the dependency on ARID1A function. Second, the timing and tissue specificity of ARID1A loss in our model driven by Pdx1‐Cre during pancreatic development may enhance the requirement for complete loss to achieve malignant transformation. Third, in humans, additional cooperating mutations or environmental factors may amplify the oncogenic effects of partial ARID1A loss, whereas our controlled genetic background in mice may require full deletion to produce a comparable phenotype. Lastly, it is possible that hemizygous ARID1A loss in humans contributes to early neoplastic changes, while biallelic inactivation is selected for during progression to more advanced or invasive disease stages.

The importance of FASN in the pathogenesis of various human diseases has been suggested recently. O'Farrell et al. demonstrated that FASN blockade directly reduced fat accumulation in liver cells and attenuated inflammation and fibrosis in animals.^29^ Additionally, FASN inhibition also affected immune and stellate cells and decreased pro‐inflammatory cytokine production and fibrotic marker expression. Thus, targeting FASN provides a new strategy for treating non‐alcoholic steatohepatitis. In small cell lung cancer, USP13 is highly expressed and is associated with poor prognosis. USP13 interacts with FASN to enhance its protein stability to promote fatty acid synthesis, cancer stemness and sphere formation.^30^ Inhibition of FASN resulted in the decrease of lipogenesis, self‐renewal ability and chemotherapy resistance in the cancer cells. In neuroblastoma, expression of FASN is correlated with worse prognosis in patients and FASN inhibitors induced neural differentiation and decreased tumour burden in a xenograft animal model.^31^ Preclinical study of FASN inhibitors in primary colon cancer cells and in a patient‐derived xenograft model of colorectal cancer also demonstrated potent anti‐tumour activity by suppressing the activation of different oncogenic pathways.^32^ These results suggested that FASN is a promising target for cancer treatment.

A novel finding in this study is the FASN‐mediated ERK activation. Previous studies showed that oncogenic KRAS and activated epidermal growth factor receptor (EGFR) promoted FASN expression via ERK in pancreatic and lung cancer cells.^33^ However, whether FASN‐induced fatty acid metabolism affects the activity of ERK has little been addressed. Two potential mechanisms could be involved in FASN‐induced ERK activation. First, increase of fatty acids by FASN may alter post‐translational modifications (like palmitoylation) of growth factor receptors to enhance ERK activity.^34^ Second, FASN‐generated fatty acids may be metabolised to other lipid signalling molecules to enhance ERK activity.^35^ Here, we clearly showed that ERK is a specific downstream mediator for FASN to promote pancreatic tumourigenesis because inhibition of FASN only selectively suppressed ERK activation while p38 activity was not affected.

The impact of ARID1A depletion in the regulation of tumour microenvironment should be emphasised. In metastatic urothelial carcinoma, the status of *ARID1A* mutation and CXCL13 expression in tumour tissues predicted the clinical responses to immune checkpoint therapy.^36^ In colorectal cancer, *ARID1A*‐mutated tumours exhibited increased tumour mutational burden, frameshift mutations and higher expression of immune checkpoint genes.^37^ Thus, microsatellite stable colorectal cancer patients with *ARID1A* mutation are more responsive to immunotherapy. In pancreatic cancer, genetic alterations in the SWI/SNF chromatin remodelling components including ARID1A is correlated with increased immunotherapy response.^38^ We found increased infiltration of various immune cells including T cells, granulocytes, neutrophils and B cells in the KAR tumours, consisting with the finding that *ARID1A*‐mutated pancreatic cancer patients are more responsive to immune checkpoint blockade.

Comparison of the microenvironment in tumours generated from different genetically engineered mice have little been addressed before. In addition, the dynamic alterations in tumour microenvironment during tumour development are also largely unclear. In this study, we characterised for the first time the dynamic changes in KAR and KPC tumours by sc‐RNA sequencing. The altered expression of key genes in immune cells confirms the impact of Arid1a deletion on immune responses within the tumour microenvironment. Collectively, our study elucidates a novel mechanism by which ARID1A loss contributes to the development of pancreatic cancer and clarify the distinct features in the microenvironment of pancreatic tumours with different genetic alterations. Moreover, we identify FASN as a new therapeutic target in ARID1A‐deficient pancreatic cancer.

## 4 EXPERIMENTAL SECTION

### 4.1 Genetically modified mice and primary culture of pancreatic cancer cells

Pdx‐1Cre, LSLKrasG12D and strain Arid1atm1.1Zhwa mice were obtained from the Mouse Models of Human Cancers Consortium (MMHCC) under material transfer agreements, and The Jackson Laboratory. Mice were genotyped as described by the MMHCC and The Jackson Laboratory (JAX) PCR protocols. Animal studies were approved by the Institutional Animal Care and Use Committee of the National Health Research Institutes. Experiments were performed on off‐spring male mice and the age of mice used in this study was from 4‐week‐old to 1‐year‐old (for medium survival determination).

### 4.2 Cell culture and primary murine pancreatic tumour cell isolation

Primary murine pancreatic cancer cells (KAR and KPC) were isolated from tumours derived from genetically engineered mouse models. Tumour tissues were finely minced and digested overnight at 37°C in Dulbecco's modified Eagle medium (DMEM) containing .012% (w/v) collagenase XI and .012% (w/v) dispase, supplemented with 1% foetal bovine serum (FBS). After digestion, the cell suspension was filtered through a 70 µm strainer, washed and cultured in RPMI‐1640 medium (Gibco) supplemented with 10% FBS, 1% penicillin–streptomycin (P/S) and 1% L‐glutamine. Human pancreatic cancer cell lines were obtained from ATCC and maintained under standard conditions. AsPC‐1 cells were cultured in RPMI‐1640 medium supplemented with 10% FBS and 1% P/S. MIAPaCa‐2 cells were cultured in DMEM high glucose supplemented with 10% FBS, 2.5% horse serum and 1% P/S. BxPC‐3 cells were cultured in RPMI‐1640 medium supplemented with 10% FBS and 1% P/S. All cells were maintained at 37°C in a humidified incubator with 5% CO₂. All media components were obtained from Gibco (Thermo Fisher Scientific) unless otherwise stated.

### 4.3 FASN inhibitor (TVB‐2640) treatment

KPC and KAR pancreatic cancer cell lines were seeded in 96‐well plates at a density of 3000–5000 cells per well and allowed to adhere overnight. Cells were then treated with a range of TVB‐2640 concentrations (20 nM to 80 µM) for 24 h. DMSO was used as the vehicle control. After treatment, cell viability was assessed using the MTT assay (Sigma‐Aldrich), following the manufacturer's protocol. Absorbance was measured at 570 nm using a microplate reader. The viability of each group was normalised to that of the DMSO‐treated control, which was set to 1.0. Each condition was tested in triplicate, and experiments were repeated at least three times independently. For in vivo treatment, TVB‐2640 was first dissolved in DMSO at a stock concentration of 50 mg/mL, and then diluted with phosphate‐buffered saline (PBS) to a final concentration of 5 mg/mL immediately before use. Six‐week‐old KAR mice were administered intraperitoneal injections of TVB‐2640 (5 mg/kg) or vehicle control twice weekly for 6 weeks (*n* = 5 per group). The vehicle solution consisted of DMSO diluted in PBS, matching the final DMSO concentration used in the drug‐treated group. At the end of the experiment, blood samples were collected, and pancreases were harvested for histological analyses.

### 4.4 Fatty acid supplementation and combination treatment with TVB‐2640

For fatty acid supplementation experiments, palmitate (Sigma‐Aldrich) was conjugated to fatty acid‐free bovine serum albumin (BSA) prior to use. A 100 mM palmitate stock was prepared in .1 M NaOH and heated to 70°C, then mixed with 10% BSA solution in a 1:4 ratio to form a palmitate–BSA complex. This complex was diluted in culture medium to the desired final concentrations of 20 nM or 50 nM. For combination treatment experiments, cells were treated with palmitate alone (20 nM or 50 nM), TVB‐2640 alone (10 µM or 40 µM) or a combination of palmitate and TVB‐2640 at the indicated concentrations. Treatments were applied for 24 h, and cell viability was assessed using the MTT assay, as described above. All conditions were tested in triplicate, and experiments were independently repeated at least three times.

### 4.5 Immunohistochemistry and immunofluorescence

Periodic acid‐Schiff stain (PAS) and Alcian blue staining kits were purchased from Scy‐Tek Laboratories and performed according to the manufacturer's protocols. The images of the IHC‐stained slides were captured using a Carl Zeiss Axioskop 2 plus microscope (Carl Zeiss). Immunofluorescence (IF) images were studied using the ECLIPSE TE2000U laser scanning confocal microscope (Nikon) and analysed using the EZ‐C1 software (Nikon). The following primary antibodies were used: Anti‐CK19 (GeneTex, GTX112666); Anti‐ARID1A (Abnova, MAB15809); Anti‐FASN (Abcam, ab22759); Anti‐CD31 (Abcam, ab28364); Anti‐ki67 (Abcam, ab15580). Masson's trichrome staining was performed on paraffin‐embedded pancreatic tissue sections using an aniline blue‐based kit (Abcam, ab150686), according to the manufacturer's instructions. Adjacent tissue sections from KAR, KAR^het^ and KPC mice were used for staining. During image quantification, empty neoplastic ducts (gland‐like structures) were carefully excluded from the total area analysed to ensure accurate assessment of stromal collagen deposition. A set of five mice was used for staining and quantification of three fields of view of pancreatic lesions.

### 4.6 Western blotting

Total proteins were extracted from cells with radioimmunoprecipitation assay (RIPA) buffer (50 mM Tris–HCl, pH 7.4, 150 mM NaCl, 1% NP‐40, .1% SDS, .5% sodium deoxycholate, 2 mM EDTA and 50 mM NaF) containing protease inhibitors. The concentrations of cellular proteins were determined by Bradford assay. Forty micrograms of cellular proteins were separated by SDS‐polyacrylamide gel electrophoresis. Proteins were transferred to polyvinylidene difluoride membranes and probed with various primary and secondary antibodies. Finally, the signals on the membranes were developed by enhanced chemiluminescence reagent.

### 4.7 Single‐cell isolation and sc‐RNAseq data analysis

To isolate cells from pancreas, KPC and KAR mice (at the age of 8 and 16 weeks) were euthanised by inspiration of 5% CO_2_. Pancreatic tissues were washed in ice‐cold PBS and manually minced into small fragments using a sterile scalpel blade. The minced tissues were then transferred into 3–5 mL of dissociation buffer from the Miltenyi Tumour Dissociation Kit (Miltenyi Biotec, Cat. No. 130‐095‐929) and further processed into submillimeter particles. The samples were enzymatically and mechanically dissociated using the gentleMACS Octo Dissociator (Miltenyi Biotec, Cat. No. 130‐095‐937) for 25–30 min. After digestion, the resulting cell suspension was filtered through a 40 µm mesh filter to remove undigested debris and obtain a single‐cell suspension. Dissociated cells were quenched with ice‐cold RPMI 1640 medium containing 10% FBS, treated with ACK lysis buffer (3–5 mL) to remove red blood cells, and then washed again with RPMI 1640 containing 10% FBS. Isolated cells were subjected to sequencing library construction and sc‐RNAseq was done as previously described.^22^ Gene‐cell matrixes were filtered to remove cells (<200 transcripts/cell, >10% mitochondria genes) and genes (<10 cells/gene). The matrix was imported into the R package Seurat (v 3.1.2) for subsequent analysis. The gene expression levels were normalised so that the number of unique molecular identifiers in each cell (UMI/cell) is equal to the median UMI. Total 2000 highly variable genes were generated for performing principle component analysis and significant principle components were determined using Jackstraw. Finally, single‐cell clustering was visualised by UMAP utilising previous computed principle components 1–10. The package (clusterProfiler) was applied to analyse and visualise functional profiles gene ontology (GO) and Kyoto Encyclopedia of Genes and Genomes (KEGG) of gene clusters. We selected .05 as the cut‐off of *p* value and *q* value.

### 4.8 Gene set variation analysis

To explore whether any gene set/biological pathway is enriched differentially between KAR and KPC samples, we used GSVA, a Gene Set Enrichment Analysis (GSEA)‐based method that estimates variation of pathway activity over a sample population in an unsupervised way. It applies the Kolmogorov–Smirnov‐like random walk statistic to assess the enrichment score (ES) of a target gene set in each cell. Each ES represents the degree to which the genes in a target gene set are co‐ordinately up‐ or downregulated within a cell.

### 4.9 Bulk RNA sequencing and data analysis

The KAR or KPC tumour tissues were minced and digested overnight with .012% (w/v) collagenase XI and .012% (w/v) dispase in DMEM media containing 1% FBS. The isolation of mouse pancreatic cancer cells was performed as described previously.^39^ Tumourigenicity of the isolated primary mouse pancreatic cancer cells was tested by injecting the cells into severe combined immunodeficient (SCID) mice and the tumours were histopathologically characterised by a pathological examination. Primary cancer cells of less than six passages were used for RNA sequencing analysis. RNA purity and quantification were checked using SimpliNano™—Biochrom Spectrophotometers (Biochrom). RNA degradation and integrity were monitored by Qsep 100 DNA/RNA Analyzer (BiOptic Inc.). One microgram of total RNA per sample was used as input material for sample preparation. Sequencing libraries were generated using KAPA mRNA HyperPrep Kit (KAPA Biosystems, Roche) following manufacturer's recommendation and the library quality was assessed on the Qubit@ 2.0 Fluorometer (Thermo Scientific) and Agilent Bioanalyzer 2100 system. The library was sequenced on an Illumina NovaSeq6000 platform and 150 bp paired‐end reads were generated. The original data were transformed into raw sequenced reads by CASAVA base calling and stored in FASTQ format. FastQC and MultiQC were used to check FASTQ files for quality control. The obtained raw paired‐end reads were filtered by Trimmomatic to discard low‐quality reads, trim adaptor sequences and eliminate poor‐quality bases. The obtained high‐quality data (clean reads) were used for subsequent analysis. Read pairs from each sample were aligned to the reference genome (e.g., *H. sapiens*, GRCh38) by the HISAT2 software. DEGs analysis of two conditions was performed in R using DEGseq (without biological replicate) and DESeq2 (with biological replicate). The resulting *p* values were adjusted using the Benjamini and Hochberg's approach for controlling the false discovery rate (FDR). GO and KEGG pathway enrichment analysis of DEGs were conducted using ClusterProfiler. GSEA was performed with 1000 permutations to identify enriched biological functions and activated pathways from the molecular signatures database (MSigDB).

### 4.10 Lentivirus production and shRNA for gene knockdown

The plasmids required for shRNA lentivirus production were purchased from the National RNAi Core Facility (Academia Sinica). The pLKO.1‐shRNA vectors used for knockdown of ARID1A were TRCN0000059090 and TRCN0000059091 (Human), TRCN0000071396 and TRCN0000071397 (Mouse). The pLKO.1‐shRNA vectors used for knockdown of FASN were TRCN0000003127 and TRCN0000003126 (Human), TRCN0000075703 and TRCN0000325036 (Mouse). The pLKO.1‐emptyT control plasmid was TRCN0000208001. To generate recombinant lentivirus, 293T cells were co‐transfected with package plasmid (pCMV8.91), envelop VSV‐G plasmid (pMD.G) and shRNA expressing construct. The virus‐containing supernatant was harvested at 48 h after transfection. Target cells were incubated with the virus‐containing medium supplemented with 8 µg/mL of polybrene and the infected cells were selected by 2 µg/mL of puromycin for 72 h before harvesting for Western blot analysis.

### 4.11 Chromatin immunoprecipitation assay

ChIP assays with the anti‐Arid1a antibody (Abnova, MAB15809) were performed using the EZ‐ChIP Kit (Merck Millipore) according to the manufacturer's instruction. Cellular lysates were subjected to five sets of sonication with a 60 Sonic Dismembrator (Fisher Scientific). Each set consisted of 8 s of sonication separated by 1‐min intervals on ice and sonicated to an average DNA length between 300 and 700 bp. Chromatin concentrations were determined using a bicinchoninic acid (BCA) protein assay kit (Pierce). Chromatin samples were diluted to 300 µg per 200 µL of nuclear lysis buffer and cleared by centrifugation at 10 000 × *g*. Precleared chromatin samples (200 µL each) were added to 800 µL of ChIP dilution buffer (50 mM Tris–Cl [pH 7.5]; 150 mM NaCl; 5 mM EDTA; .5% IGEPAL CA‐630; 1% TX‐100) and incubated with 10 µg of prebound anti‐ARID1A Protein G beads (Invitrogen) overnight at 4°C. The immunoprecipitated complexes were washed twice with 1 mL ChIP dilution buffer, three times with 1 mL ChIP dilution buffer supplemented with 500 mM NaCl, twice with 1 mL ChIP dilution buffer, followed by a final wash in 1 mL low‐salt Tris‐EDTA (TE) buffer (10 mM Tris–Cl [pH 8.0]; 1 mM EDTA; 50 mM NaCl). The immunoprecipitated samples were eluted in 100 µL of elution buffer (50 mM NaHCO_3_; 1% SDS) at 62°C for 20 min. The crosslinks were reversed by adding NaCl to a final concentration of .2 M followed by an incubation at 65°C. The samples were deproteinated with Proteinase K and purified using the ChIP DNA Clean & Concentrator kit (Zymo Research) according to the manufacturer's Instructions. Precipitated DNA was analysed using PCR primers for Fasn promoter listed in Table S1. Real‐time quantitative PCR using Ssofast PCR master mix (Biorad) and a IQ5 thermocycler (Biorad).

### 4.12 RNA sequencing of PDAC specimens

RNA sequencing analysis of 105 pancreatic ductal adenocarcinoma (PDAC) specimens was performed and this study was approved by the Institutional Review Board of the National Cheng Kung University Hospital (B‐ER‐110‐420). Patient anonymity was preserved. Libraries were constructed and loaded onto Illumina NovaSeq system (Illumina) and sequencing was performed using a 2 × 150 paired‐end configuration.

### 4.13 Quantification and statistical analysis

All experiments were performed at least three times in triplicate, and representative data were shown. Statistical analysis was performed by one‐ or two‐way ANOVA using Prism 5.0 software to identify differences among different experimental groups. The median survival was estimated using the Kaplan–Meier method. Quantitative data were expressed as mean ± SEM and *p* value less than .05 was considered significant. The significance was presented as *p < .05; **p < .01; ***p < .001; and no significant difference was presented as ns.

## AUTHOR CONTRIBUTIONS

Tzu‐Lei Kuo performed the experiments; Tzu‐Lei Kuo, Ya‐Chin Hou and Wen‐Chun Hung analysed the data; Ya‐Chin Hou and Yan‐Shen Shan provided critical samples/materials and reagents/protocols; Tzu‐Lei Kuo and Wen‐Chun Hung wrote and edited the manuscript. Yan‐Shen Shan, Li‐Tzong Chen and Wen‐Chun Hung conceived the project idea.

## CONFLICT OF INTEREST STATEMENT

The authors declare no conflicts of interest.

## ETHICS STATEMENT

Animal studies were approved by the Institutional Animal Care and Use Committee of the National Health Research Institutes. RNA sequencing analysis of PDAC specimens was approved by the Institutional Review Board of the National Cheng Kung University Hospital (B‐ER‐110‐420).

## Supporting information



Supporting Information

Supporting Information

Supporting Information

Supporting Information

Supporting Information

Supporting Information

Supporting Information

Supporting Information

## Data Availability

The data that support the findings of this study are available from the corresponding author upon reasonable request.
