# Supplementary figures and images for: Arid1a deficiency sensitises pancreatic cancer to fatty acid synthase inhibition
Source: Clin Transl Med. 2025 Jul 7;15(7):e70394. doi: 10.1002/ctm2.70394 (PMC12230631; doi:10.1002/ctm2.70394)

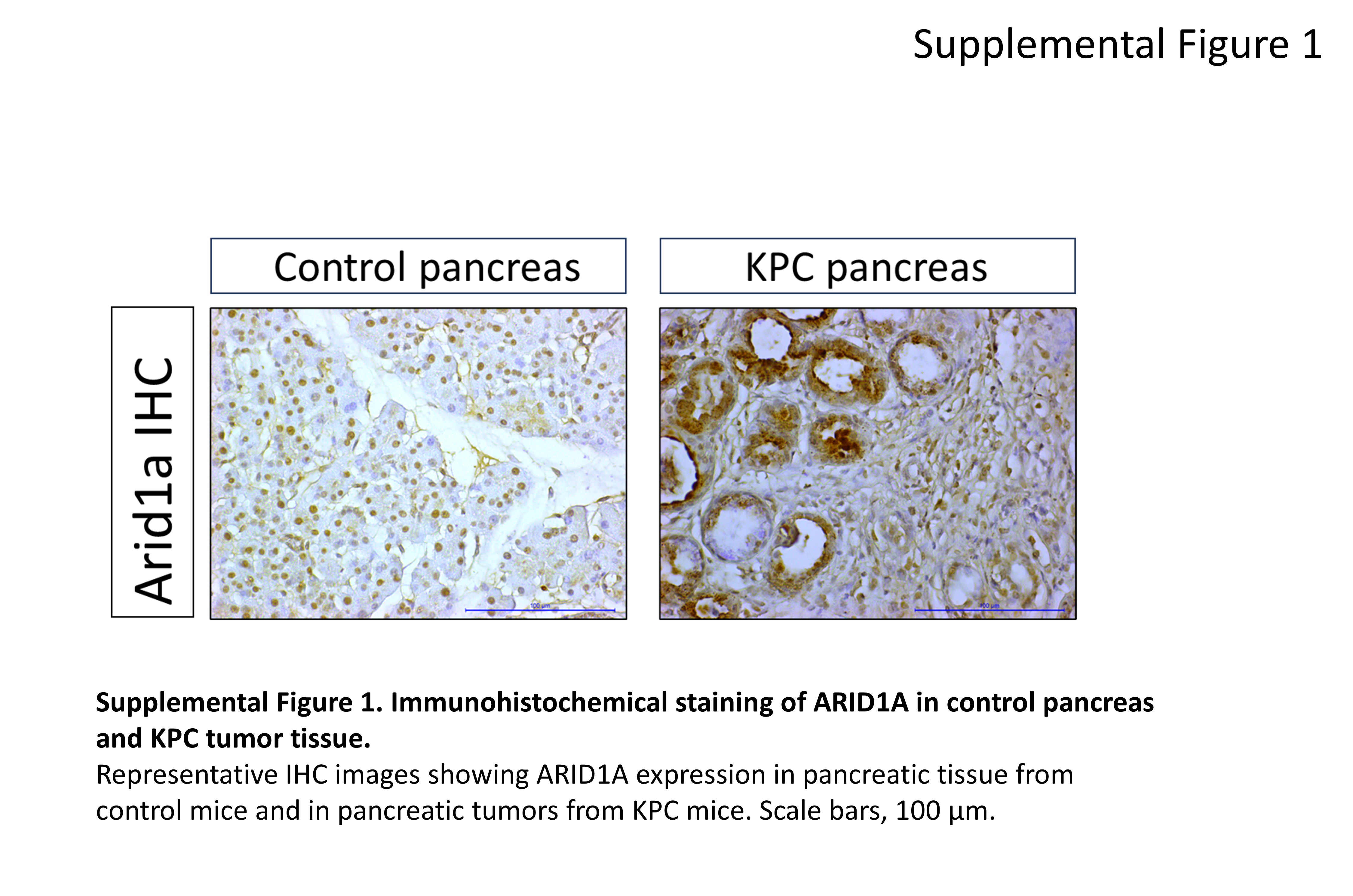

Supplement: Supplementary file 1 — Supporting Information [file CTM2-15-e70394-s003.TIF]

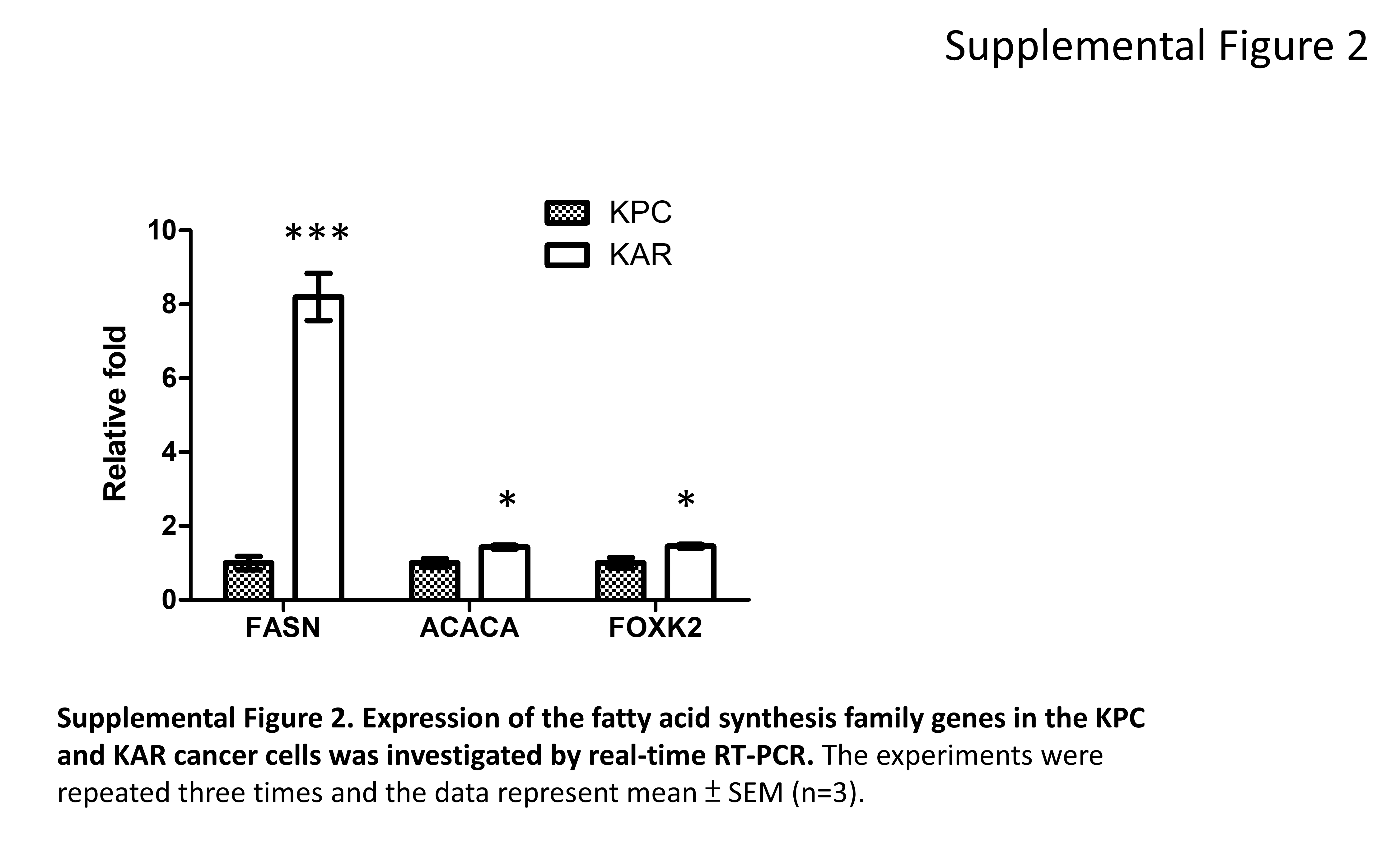

Supplement: Supplementary file 2 — Supporting Information [file CTM2-15-e70394-s004.TIF]

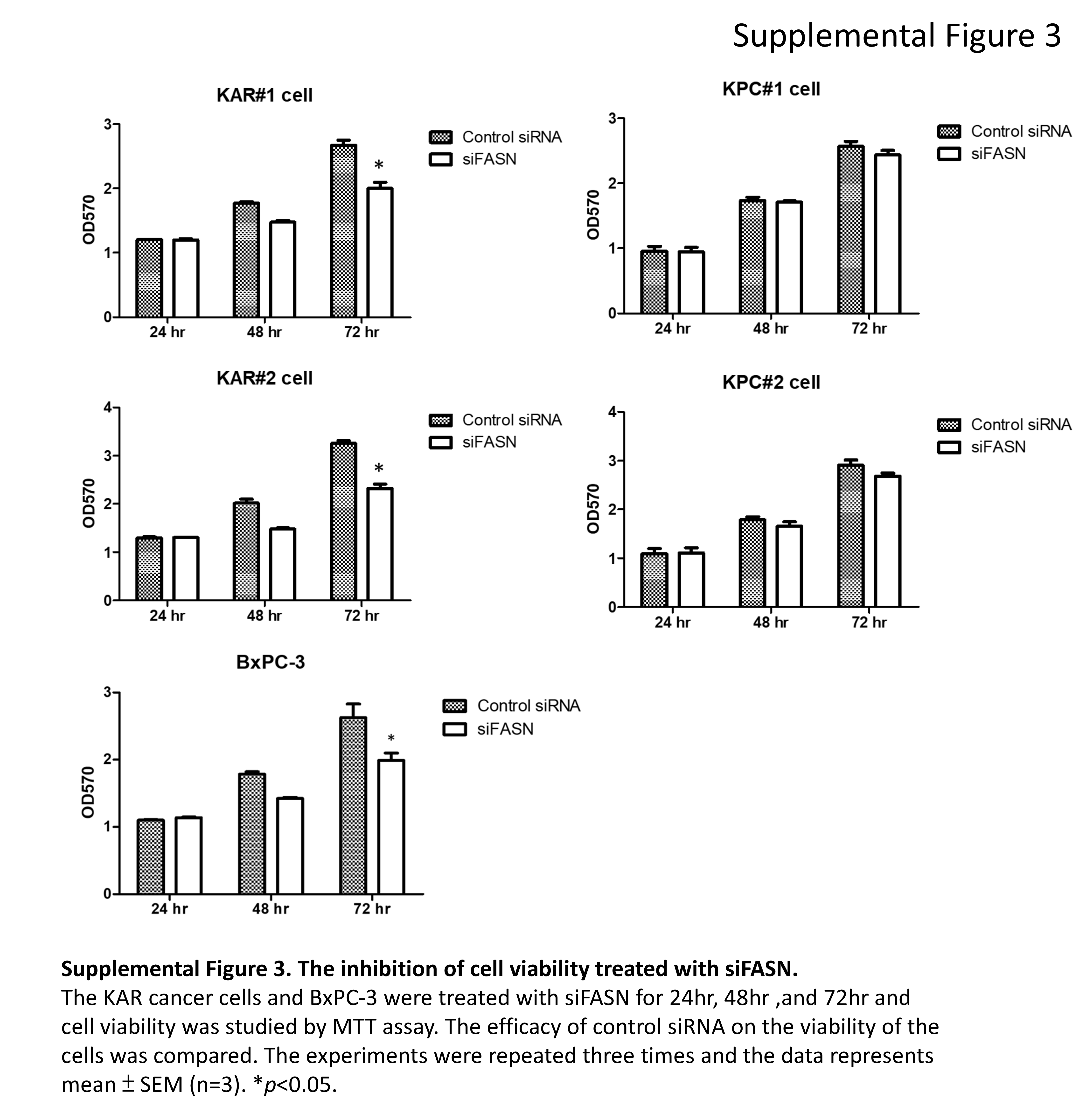

Supplement: Supplementary file 3 — Supporting Information [file CTM2-15-e70394-s006.TIF]

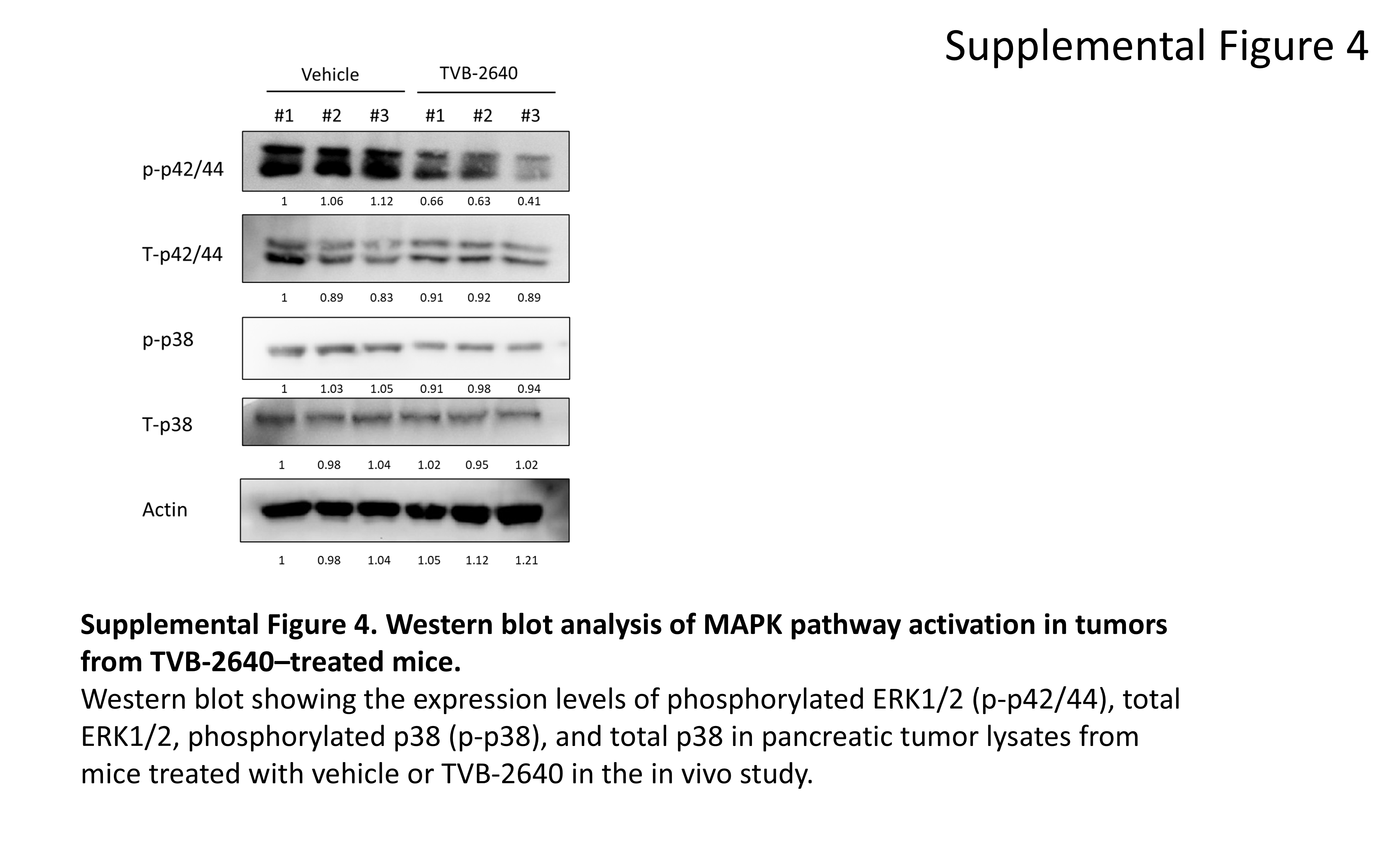

Supplement: Supplementary file 4 — Supporting Information [file CTM2-15-e70394-s002.TIF]

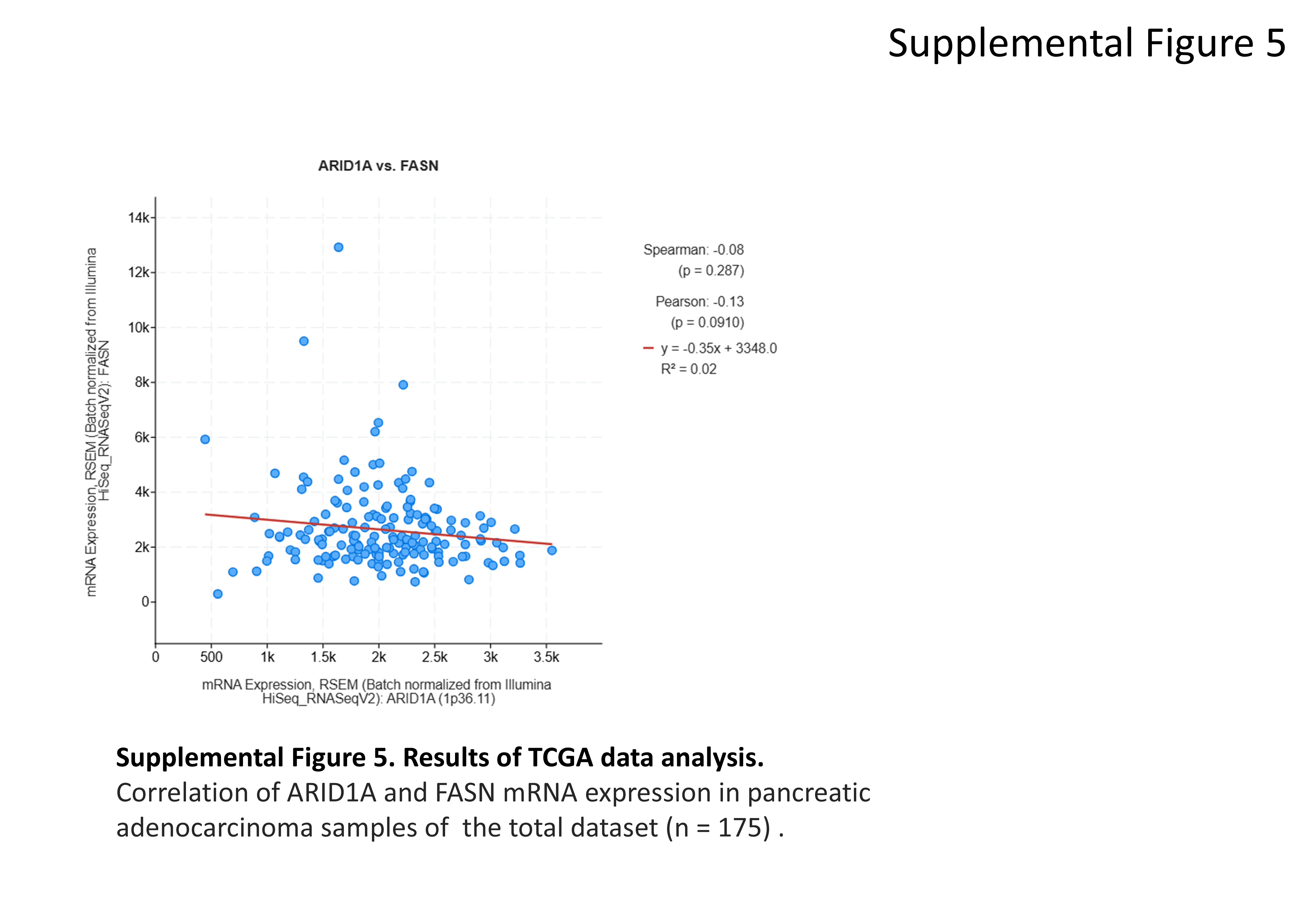

Supplement: Supplementary file 5 — Supporting Information [file CTM2-15-e70394-s008.TIF]

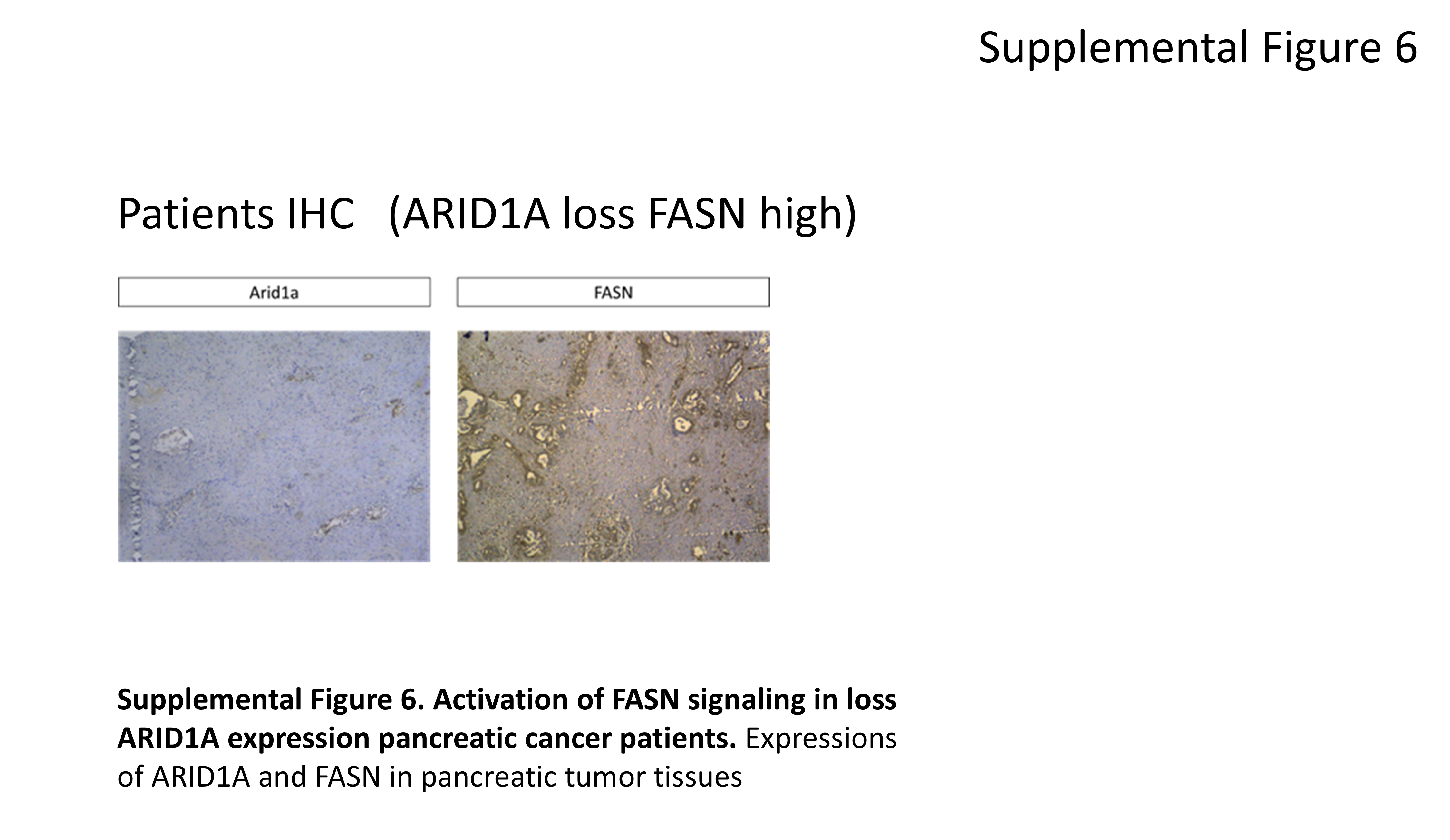

Supplement: Supplementary file 6 — Supporting Information [file CTM2-15-e70394-s001.TIF]

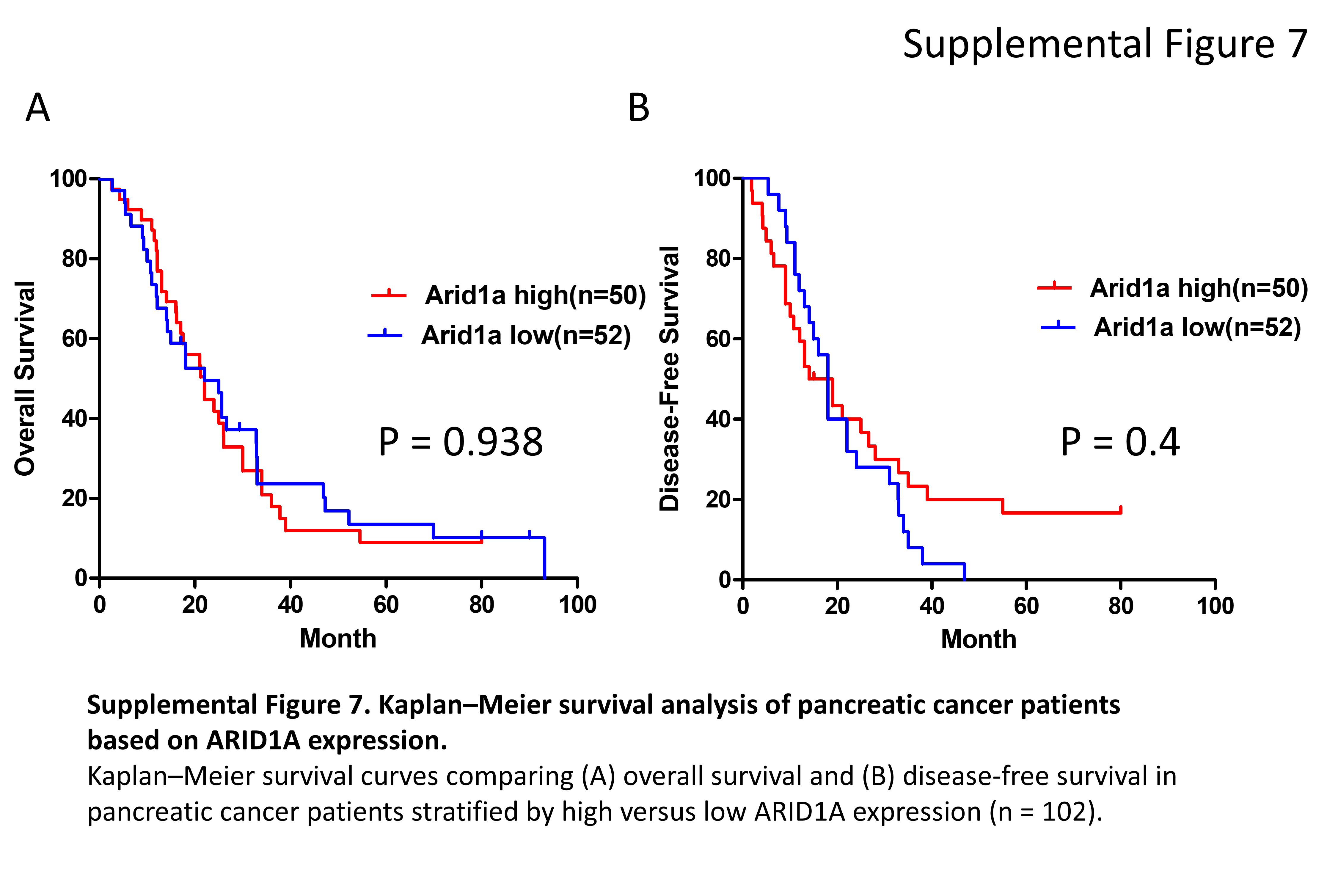

Supplement: Supplementary file 7 — Supporting Information [file CTM2-15-e70394-s007.TIF]
