# Supplementary material for: Arid1a deficiency sensitises pancreatic cancer to fatty acid synthase inhibition
Source: Clin Transl Med. 2025 Jul 7;15(7):e70394. doi: 10.1002/ctm2.70394 (PMC12230631; doi:10.1002/ctm2.70394)
Supplement: Supplementary file 8 — Supporting Information [file CTM2-15-e70394-s005.docx]

| **Supplemental Table 1**  List of the PCR primers for ChIP sequencing of Fasn promoter. | | |
| --- | --- | --- |
| Primer name | Sequence | Bases |
| FASN-F1 | CAGCCCTCCATGCTGAAACA | 20 |
| FASN-R1 | GTATTCCCCACAAGTGGCCT | 20 |
| FASN-F2 | ACACCCTCCAAGGAGTCTCA | 20 |
| FASN-R2 | TCTGCAGCTGTCAGTGTGAA | 20 |
| FASN-F3 | GTCTGCTGGGGTCCTTGTTC | 20 |
| FASN-R3 | AGCCTTTTCTACCGTGTGGC | 20 |
| FASN-F4 | GGGTGTCTCCAAGTCAGTGG | 20 |
| FASN-R4 | CACCCTGAGTGCAGGAACTG | 20 |
